# Non-Opioid Analgesics and Adjuvants after Surgery in Adults with Obesity: Systematic Review with Network Meta-Analysis of Randomized Controlled Trials

**DOI:** 10.3390/jcm13072100

**Published:** 2024-04-03

**Authors:** Michele Carron, Enrico Tamburini, Federico Linassi, Tommaso Pettenuzzo, Annalisa Boscolo, Paolo Navalesi

**Affiliations:** 1 Department of Medicine—DIMED, Section of Anesthesiology and Intensive Care, University of Padova, Gallucci V. St. 13, 35121 Padova, Italy; annalisa.boscolobozza@unipd.it (A.B.); paolo.navalesi@unipd.it (P.N.); 2 Institute of Anesthesia and Intensive Care, Padua University Hospital, Giustiniani St. 2, 35128 Padova, Italy; enrico.tamburini@aopd.veneto.it (E.T.); tommaso.pettenuzzo@aopd.veneto.it (T.P.); 3 Department of Anesthesia and Intensive Care, Ca’ Foncello Treviso Regional Hospital, Hospital Sq. 1, 31100 Treviso, Italy; federico.linassi@gmail.com

**Keywords:** obesity, anesthesia, analgesia, surgery, combined-modality therapy, treatment outcome, postoperative pain, postoperative nausea and vomiting, complications, postoperative recovery

## Abstract

**Background/Objectives:** Managing postoperative pain in patients with obesity is challenging. Although using a combination of pain relief methods is recommended for these patients, the true effectiveness of various intravenous non-opioid analgesics and adjuvants in multimodal anesthesia needs to be better defined. **Methods:** A systematic review and network meta-analysis was performed to evaluate the efficacy of nonsteroidal anti-inflammatory drugs (NSAIDs), acetaminophen, ketamine, α-2 agonists, lidocaine, magnesium, and oral gabapentinoids in adult surgical patients with obesity. The analysis aimed to compare these treatments to a placebo/no treatment or alternative analgesics, with a primary focus on postoperative pain and secondary endpoints including rescue analgesia, postoperative nausea and vomiting (PONV), and recovery quality. English-language randomized controlled trials across PubMed, Scopus, Web of Science, CINAHL, and EMBASE were considered. Quality and evidence certainty were assessed with the RoB 2 tool and GRADE, and data was analyzed with R software. **Results:** NSAIDs, along with acetaminophen, lidocaine, α-2 agonists, ketamine, and oral gabapentinoids, effectively reduce early postoperative pain. NSAIDs, particularly ibuprofen, as well as acetaminophen, ketamine, and lidocaine, also show benefits in later postoperative stages. Intravenous non-opioid analgesics and adjuvants show some degree of benefit in reducing PONV and the need for rescue analgesic therapy when using α-2 agonists alone or combined with oral gabapentinoids, notably decreasing the likelihood of PONV. Ketamine, lidocaine, and α-2 agonists are shown to enhance postoperative recovery and care quality. **Conclusions:** Intravenous non-opioid analgesics and adjuvants are valuable in multimodal anesthesia for pain management in adult surgical patients suffering from obesity.

## 1. Introduction

Managing postoperative pain in patients with obesity remains a significant challenge in surgical practice [1]. The global rise in obesity rates entails an increase in surgical patients and the need to adopt an analgesic strategy that meets the specific physiological and pharmacological needs of this patient population [1,2]. The shift towards multimodal general anesthesia [3] represents a strategic move to enhance analgesic techniques and decrease dependency on opioids, which carry risks of adverse effects and addiction [1,2]. This approach utilizes various agents targeting different components of the nociceptive pathway, promising more effective and safer pain management [3].

High-quality evidence supports the use of multimodal analgesia for postoperative pain in the general population [4]. Evidence underscores a shift towards multimodal anesthesia in surgical patients with obesity [1,2,5,6]. The role of multimodal analgesia, which integrates various drugs and techniques to enhance pain management while reducing opioid-related side effects, becomes critically significant in the context of obesity due to its unique postoperative pain management challenges [1,2,5,6]. These challenges are primarily attributed to the physiological alterations in obesity, particularly affecting airway and lung function, thereby escalating the risk of opioid-induced respiratory complications [1,2]. Consequently, an analgesic regimen that aims to minimize opioid use in the postoperative phase is recommended for these patients, a stance that is supported by both international [5] and national [6] guidelines. These guidelines endorse a reduction in opioid consumption by employing a multimodal approach [3,4,5,6]. Locoregional anesthesia, as a complement to general anesthesia, is encouraged to bolster the benefits of multimodal analgesics [1,2]. For adults suffering from obesity, employing techniques like the transversus abdominis plane block, which has proven superior in lowering opioid consumption, pain, postoperative nausea and vomiting (PONV), and the need for rescue analgesics in bariatric surgery [6,7], along with non-opioid analgesics and adjuvants such as intravenous acetaminophen, nonsteroidal anti-inflammatory drugs (NSAIDs), ketamine, α-2 agonists, lidocaine, magnesium, and oral gabapentinoids [1,2,3], is recommended to enhance pain management and reduce opioid-related adverse effects [1,5,6].

This research aims to conduct a comprehensive systematic review and network meta-analysis to assess the comparative efficacy of various intravenous non-opioid analgesics and adjuvants used in multimodal anesthesia strategies, both as standalone options and in combination, with a specific focus on managing postoperative pain in obese patients [3]. The primary objective of this research is to evaluate the effectiveness of intravenous non-opioid agents and adjuvants within a multimodal anesthesia framework in reducing postoperative pain scores, offering a quantitative analysis of their impact on pain management. Additionally, it will assess the efficacy of these non-opioid options throughout the postoperative period by examining their influence on the need for supplementary pain medication and on the incidence of common postoperative complications, such as PONV. This study will also consider the overall quality of recovery following surgery, providing a comprehensive view of the benefits of integrating intravenous non-opioid agents and adjuvants into postoperative pain management strategies.

## 2. Materials and Methods

The network meta-analysis protocol was registered prospectively under the PROSPERO identification number CRD42023399373. In the preparation of this manuscript, we ensured compliance with the Preferred Reporting Items for Systematic Reviews and Meta-Analyses (PRISMA) Statement to facilitate transparent and comprehensive reporting of our review process and findings [8].

### 2.1. Eligibility Criteria

The criteria for inclusion in this systematic review and network meta-analysis were defined according to the PICOS framework as follows:

- Population (P): The population of interest includes adult patients (aged ≥ 18 years) with obesity, defined as having a Body Mass Index (BMI) of ≥30 kg/m^2^, who are undergoing surgery.
- Intervention (I): The interventions under evaluation include multimodal, non-opioid analgesic approaches within the context of a standard anesthesiological strategy. These include the use of non-opioid analgesics and adjuvants utilized in multimodal general anesthesia, such as acetaminophen (or paracetamol), NSAIDs, ketamine, α-2 agonists (i.e., dexmedetomidine, clonidine), lidocaine, magnesium, and oral gabapentinoids (i.e., pregabalin, gabapentin) [3]. This study will consider these interventions both individually and in various combinations.
- Comparison (C): The comparator groups in this study consist of a placebo, no intervention, or alternative multimodal analgesic strategies, employed either as single agents or in combination.
- Outcomes (O): The primary outcome of interest in this study is the level of postoperative pain, which is assessed using standardized tools such as the Visual Analogue Scale (VAS) or the Numerical Rating Scale (NRS). The VAS is typically a 10 cm line ranging from “no pain” to “worst pain imaginable”, where patients mark their pain level. The NRS, on the other hand, asks patients to rate their pain on a scale usually from 0 (“no pain”) to 10 (“worst pain possible”), allowing for a numerical assessment of their pain intensity. Both scales are widely used in clinical settings for their simplicity and effectiveness in pain evaluation. Starting from the first reported time-point for the primary outcome, evaluation was extended as long as feasible to explore potential impacts not only in the immediate but also in the late postoperative period. Secondary outcomes encompass the requirement for rescue analgesic medication, the occurrence of PONV, and the assessment of post-surgical recovery quality utilizing the Quality of Recovery-40 (QoR-40) questionnaire. The QoR-40 is a detailed survey that captures various aspects of a patient’s recovery experience following surgery and anesthesia [9].
- Study Design (S): Eligible studies for this review are prospective randomized controlled clinical trials (RCTs) published in the English language and involving adult surgical patients.

Studies excluded from this review include observational studies, non-clinical research, pediatric studies, studies lacking sufficient data or a full-text version, and non-peer-reviewed articles.

### 2.2. Search Strategy

A comprehensive literature search was conducted to identify relevant studies for inclusion in this systematic review and network meta-analysis. The databases queried included PubMed, Scopus, Web of Science, CINAHL, and EMBASE, with the search being carried out up to 28 September 2023. The search strategy employed a combination of Medical Subject Headings (MeSHs) and key terms using the Boolean operators “AND” and “OR”. The MeSH terms “obesity” OR “morbid obesity” OR “bariatric surgery” were combined with “AND” to include non-opioid analgesics and adjuvants commonly used for pain management within the framework of multimodal analgesia. These included “acetaminophen” [or “paracetamol”], “NSAIDs”, “ketamine”, “α-2 agonists” (dexmedetomidine, clonidine), “lidocaine”, “magnesium”, and “pregabalin/gabapentin”, employed both singularly and in various combinations [3]. To further enhance the accuracy and comprehensiveness of the search, the reference lists of all evaluated studies were also meticulously examined. This strategy aimed to identify additional studies that may not have been captured through database searching alone. The detailed search strategy, including the specific combinations of terms and filters used, is thoroughly documented in the Supplementary Materials Content (SMC)1.

### 2.3. Study Selection, Data Extraction and Data Retrieval

Two authors independently screened the titles and abstracts of articles retrieved by the search strategies based on MeSH terms [ET, FL] and excluded non-relevant articles. The full texts of the remaining studies were then assessed to determine whether they met the pre-determined selection criteria. Data were extracted independently by two authors [ET, FL] using pre-designed data collection forms for each study. An author not involved in the literature search [MC] resolved any discrepancies that arose during the study selection, data extraction, or trial evaluation process. Two authors not previously involved in the search and data extraction process (AB, TP) manually reviewed and assessed each of the included studies, evaluated the data extracted and confirmed the final dataset. Corresponding authors of included articles were contacted by email for additional data.

### 2.4. Quality Assessment and Certainty of Evidence Assessment

A panel of authors, including those involved in the literature search, independently assessed the quality of the included RCTs using the Risk of Bias (RoB) 2 tool [10]. The RoB 2 tool assesses five key domains of risk bias: the randomization process, deviations from intended interventions, missing outcome data, the measurement of the outcome, and the selection of the reported result. Within each domain, a series of questions (“signaling questions”) are asked to gather information about potential sources of bias. Based on the responses to these signaling questions, an algorithm determines a proposed assessment of bias for each domain, ranging from “low” or “high” risk to “some concerns” [10]. Any discrepancies in the initial assessments were resolved through discussion with a third author (MC). To assess the certainty of evidence related to the outcomes, the Grades of Recommendation, Assessment, Development, and Evaluation (GRADE) approach was employed, categorizing evidence into high, moderate, low, or very low quality of evidence (QoE) [11]. The initial QoE rating is set as high for evidence from randomized controlled trials. However, this quality may be downgraded due to several factors: risk of bias (e.g., inadequate blinding or allocation concealment), inconsistency (evaluated by a variance in effect estimates across studies using measures of statistical heterogeneity such as I-squared (I^2^), tau [τ], and tau-squared [τ^2^]), indirectness (e.g., when study populations, interventions, or outcomes differ from those of primary interest), imprecision (evidenced by wide 95% confidence intervals or estimates near a null effect), and publication bias [11]. In addition to the QoE assessment, we employed the Surface Under the Cumulative Ranking (SUCRA) methodology to evaluate the impact of intravenous analgesics and adjuvants on pain relief at different time points and for other outcomes. To visually represent and analyze the data, we utilized network graphs, forest plots, rankograms, and heat maps. These tools facilitated a comprehensive understanding of the comparative efficacy and ranking of the treatments, providing clear, visual insights into our findings.

### 2.5. Statistical Analysis

The network meta-analysis was conducted within a frequentist framework. For continuous outcome data, the mean difference (MD) and 95% confidence interval (CI) were computed. For binary outcome data, we calculated the odds ratio (OR) and 95% CI. A Shapiro–Wilk test for normality was applied to the continuous data when the number of combined studies exceeded three. In cases where studies reported a median and interquartile range, these were converted to estimated mean and standard deviation (SD) using Hozo’s method [12]. Both random and fixed effects models were employed for meta-analyses. For both dichotomous and continuous data, effects were computed using the inverse variance method, which has the advantage of also providing results for random effects. In the case of dichotomous data, in calculating the OR, an adjustment of adding 0.5 was made to the frequencies in studies reporting zero events. The Mantel–Haenszel approach was compared with the inverse variance method, leading to very similar results. The DerSimonian and Laird method was employed for inverse-variance weighting to accommodate heterogeneity. Heterogeneity among studies was evaluated using the I^2^ measure, with a threshold of *p* < 0.1 set to determine its presence. I^2^ values were categorized as low (<25%), moderate (25–50%), or high (>50%) [13]. To further quantify heterogeneity, τ was calculated to assess the standard deviation of underlying effects across studies, indicating the degree of variation beyond chance, and τ^2^ was estimated, providing a measure of the between-studies variance. In the case of significant heterogeneity among study outcomes, the random effects model was preferred, providing a more accurate reflection of the data variability across different study settings. In situations where the number of included studies was limited, accurately estimating τ^2^ became challenging yet essential for interpreting heterogeneity. The presence of significant heterogeneity was also supported by Q test results, guiding the interpretation and application of the meta-analysis findings. Funnel plots were utilized for visual inspection to assess the risk of publication bias in meta-analyses, whereas Egger’s test for asymmetry was applied exclusively to analyses comprising 10 or more studies. A *p* < 0.1 suggests a possible risk of publication bias, whereas a *p* ≥ 0.1 indicates no substantial risk of publication bias [14]. All analyses were conducted using R software, version 4.3.1 (2023). The “*netmeta*” library was specifically employed for conducting a network meta-analysis. Consistent with standard statistical practices, all *p*-values were two-tailed, with a significance threshold set at <0.05.

## 3. Results

### 3.1. Paper Selection

**Figure 1:**
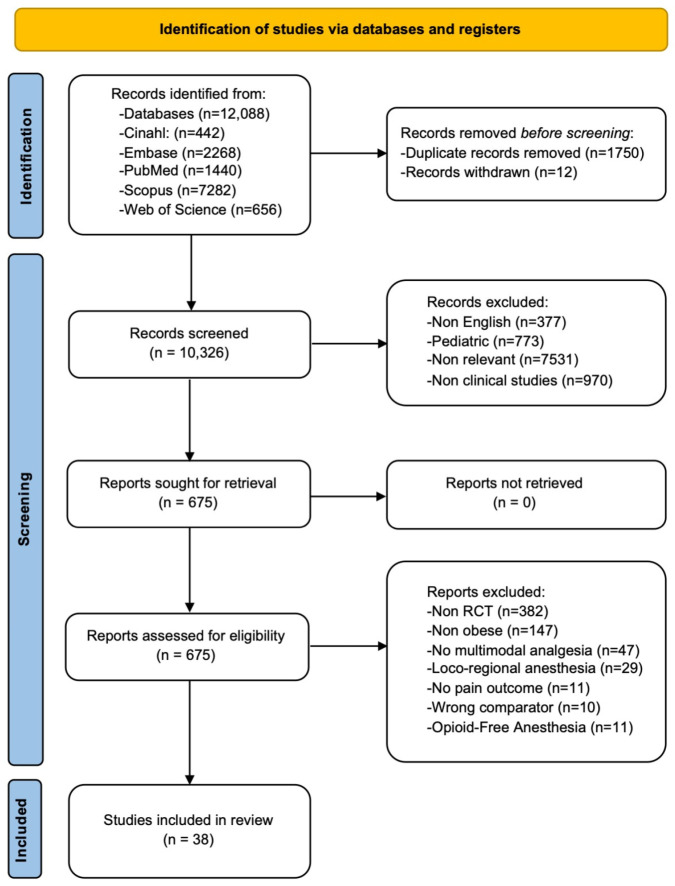
The PRISMA flow diagram of the study selection process.

Of the 12,088 reports initially identified for screening in the literature, 12,050 records were excluded because they did not meet the inclusion criteria. Therefore, 38 RCTs involving a total of 3570 patients were eligible for the network meta-analysis [15,16,17,18,19,20,21,22,23,24,25,26,27,28,29,30,31,32,33,34,35,36,37,38,39,40,41,42,43,44,45,46,47,48,49,50,51,52]. The PRISMA flow diagram of our study selection protocol is presented in Figure 1.

### 3.2. Study Characteristics

The characteristics of the included RCTs [15,16,17,18,19,20,21,22,23,24,25,26,27,28,29,30,31,32,33,34,35,36,37,38,39,40,41,42,43,44,45,46,47,48,49,50,51,52] are available for consultation in SMC2. An analysis shows that out of the total participants, 1795 patients were allocated to the control (1491 placebo or no intervention and 304 to a comparator), while 1775 were allocated to treatment (135 to Ibuprofen [28,33], 207 to acetaminophen (or paracetamol) [25,27,29,33], 329 to ketamine [16,20,37,39,40,42,43,45,49,50,52], 23 to ketamine plus clonidine [18], 68 to ketamine plus magnesium [39,42], 391 to dexmedetomidine [15,17,22,26,31,36,47,49], 341 to lidocaine [21,26,38,41,44,46,47,51], 70 to magnesium [34,48], 112 to gabapentin [23,32,35], 69 to pregabalin [19,30], and 30 to pregabalin plus dexmedetomidine [24]). Regarding the comparison between treatment and the comparator, acetaminophen was compared with ibuprofen in 89 patients [28,33], clonidine with dexmedetomidine in 30 patients [22], ketamine with dexmedetomidine in 54 patients [47,49], lidocaine in 24 patients [47], ketamine plus magnesium in 37 patients [39,42], and lidocaine with dexmedetomidine in 70 patients [26,47].

### 3.3. Risk of Bias Assessment

**Figure 2:**
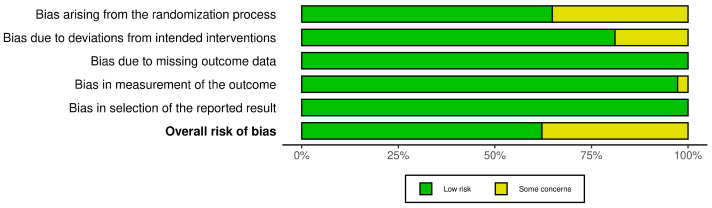
Summary plot of the RoB 2 assessment for the included RCTs.

The RoB 2 assessment for the included RCTs indicated that the studies exhibit a low or unclear risk of bias [15,16,17,18,19,20,21,22,23,24,25,26,27,28,29,30,31,32,33,34,35,36,37,38,39,40,41,42,43,44,45,46,47,48,49,50,51,52]. The distribution of risk-of-bias judgments across different domains is depicted in the weighted bar plots in Figure 2, with 35.1% of studies raising “some concerns” in the domain related to the randomization process and 18.9% in the domain related to deviations from intended interventions. The “traffic light” plots, providing a detailed view of the domain-level judgments for each individual study, are presented in SMC3. Overall, all studies provided information about randomization, but some studies were unclear about the randomization process or did not specify the allocation concealment or masking strategy used [15,16,18,19,20,22,23,30,32,39,42,44,47]. Some studies did not specify the method for blind operators and participants [15,18,20,30,39,44]. All studies reported outcome data according to the endpoint of the study [15,16,17,18,19,20,21,22,23,24,25,26,27,28,29,30,31,32,33,34,35,36,37,38,39,40,41,42,43,44,45,46,47,48,49,50,51,52]. In only one study was it declared that the outcome assessor was aware of the intervention received by the study participants [18], while in one study it was not clearly specified [44]. The risk of reporting bias was low in all studies [15,16,17,18,19,20,21,22,23,24,25,26,27,28,29,30,31,32,33,34,35,36,37,38,39,40,41,42,43,44,45,46,47,48,49,50,51,52]. Outcome measurements and analyses were conducted in accordance with a prespecified plan to eliminate the possibility of bias in result selection [15,16,17,18,19,20,21,22,23,24,25,26,27,28,29,30,31,32,33,34,35,36,37,38,39,40,41,42,43,44,45,46,47,48,49,50,51,52]. The detailed reasons for the risk of biased judgments are available in SMC4.

### 3.4. Outcomes

Network graphs related to the study outcomes are provided in SMC5. Forest plots for the network meta-analysis are available in SMC6, displaying the estimated effects of treatments and their confidence intervals. The results and SUCRA rankings for all outcomes are presented in Table 1 and Table 2 and are visualized as graphs in SMC7 (Figures S1–S3). The funnel plots for the network meta-analysis, evaluating the potential presence of risk of publication bias, are provided in SMC8. Rankograms from the network meta-analysis, which illustrate the probability distribution of treatment rankings for effectiveness across different outcomes, are presented in SMC9. Heat maps from the separate indirect from direct evidence (SIDE) analysis, which help to evaluate the consistency between direct and indirect evidence within the network meta-analysis, are reported in SMC10.

#### 3.4.1. Postoperative Pain

Postoperative pain was evaluated at different time points postoperatively, at the end of anesthesia [17,18,22,23,27,30,33,35,38,42,47,49], 30 min [17,18,31,40,48,49,50], 60 min [15,17,18,20,23,30,31,38,40,47,49,50,51], 2 h [15,20,23,31,33,38,40,48,49,50,51], 4 h [23,32,33,35,36,38,42,48,51], 6 h [18,22,23,31,38,47,49,50], 8 h [33,35,42,48], 12 h [18,22,31,32,33,38,42,45,47,49,50], 24 h [17,22,25,27,28,29,30,31,33,35,38,42,43,44,45,47,49,50,52], 48 h [17,27,44,45,47,50,52] after surgery, and at 7 days post-surgery [17,52] (Table 1). No evaluations for the specified outcomes are available beyond 7 days post-surgery. Overall, NSAIDs significantly reduced the pain score at the end of anesthesia (MD: −3.27) [33], 2 h (MD: −2.37) [33], 4 h (MD: −1.63) [33], 8 h (MD: −2.50) [33], 12 h (MD: −2.23) [33], and 24 h (MD: −1.0) [28,33] after surgery (Table 1). Acetaminophen (or paracetamol) significantly reduced the pain score at the end of anesthesia (MD: −1.88) [27,33], 2 h (MD: −1.53) [33], 8 h (MD: −1.8) [33], and 12 h (MD: −1.63) [33] after surgery (Table 1). Ketamine significantly reduced the pain score at 60 min (MD: −1.31) [20,40,49,50], 2 h (MD: −1.03) [20,40,49,50], and 12 h after surgery (MD: −0.43) [42,45,49,50] (Table 1). Dexmedetomidine significantly reduced the pain score at 30 min (MD: −1.36) [17,31,49], 60 min (MD: −1.24) [15,17,31,47,49], 2 h (MD: −0.87) [15,31,49], 4 h (MD: −3.10) [36], 6 h (MD: −0.44) [22,31,47,49], and 48 h (MD: −0.80) [17,47] after surgery (Table 1). Clonidine reduced the pain score 6 h after surgery (MD: −1.73) [22]. Clonidine combined with ketamine significantly reduced the pain score at the end of anesthesia (MD: −2.0) [18] (Table 1). Lidocaine significantly reduced the pain score at the end of anesthesia (MD: −2.16) [38,47], 60 min (MD: −2.23) [38,47,51], 6 h (MD: −2.27) [38,47], and 12 h (MD: −1.90) [38,47] after surgery (Table 1). Magnesium significantly reduced the pain score 4 h after surgery (MD: −2.06) [48] (Table 1). Gabapentin significantly reduced the pain score at the end of anesthesia (MD: −2.65) [23,35], 4 h (MD: −1.19) [23,32,35], and 8 h (MD: −2.44) [35] after surgery (Table 1). Pregabalin did not significantly impact the pain score after surgery (Table 1). According to SUCRA rankings, the most effective drugs for reducing postoperative pain scores were NSAIDs at the end of anesthesia, dexmedetomidine at 30 min, lidocaine at 60 min, NSAIDs at 2 h, dexmedetomidine at 4 h, lidocaine at 6 h, NSAIDs at 8, 12 and 24 h, and dexmedetomidine at 48 h after surgery (Table 1, Figure S1). No medication was shown to significantly alter the pain score 7 days after surgery (Table 1), and no data are available for time points beyond this. The SIDE heatmap for the VAS at 60 min post-surgery highlights inconsistencies in lidocaine comparisons [SMC10]. The QoE for the outcomes measured is detailed in Table 1. The study analysis at various postoperative time points contributed to the following QoE assessments for VAS measurements: at the end of surgery: 35.7% of studies contributed to a moderate QoE and 64.3% to a low QoE; at 30 min, contributions were 55.6% to moderate and 44.4% to low; at 60 min, 87% contributed to moderate and 13% to low; at 2 h, contributions were 75% to moderate and 25% to low; at 4 h, 53.8% contributed to moderate and 46.2% to low; at 6 h, 56.3% contributed to moderate and 43.8% to low; at 8 h, the contribution was evenly split at 50% to both moderate and low; at 12 h, 56% contributed to moderate and 44% to low; at 24 h, contributions were 9.7% to moderate and 90.3% to low; at 48 h, 35.7% contributed to moderate and 64.3% to low; and at postoperative day 7, the contribution was 0% to moderate and 100% to low.

**Table 1:** Effects of intravenous non-opioid agents and adjuvants on postoperative pain.

VAS at the End of Surgery	τ ^2^ = 0.4460; τ = 0.6678; I^2^ = 76% [58.0%; 86.3%]; *p* < 0.001 at Q Test						
Drug	MD	95% CI	z	*p* Value	Rank	P Score	QoE
**Ibuprofen**	**−3.27**	**[−4.39; −2.16]**	**−5.79**	**<0.001**	**1**	**0.954**	⊕⊕⊕⊖ Moderate ^§^
**Gabapentin**	**−2.65**	**[−3.75; −1.55]**	**−4.74**	**<0.001**	**2**	**0.838**	⊕⊕⊕⊖ Moderate ^§^
**Lidocaine**	**−2.16**	**[−3.44; −0.88]**	**−3.31**	**<0.001**	**3**	**0.727**	⊕⊕⊕⊖ Moderate ^§^
**Ketamine + clonidine**	**−2.00**	**[−3.33; −0.66]**	**−2.94**	**0.003**	**4**	**0.679**	⊕⊕⊕⊖ Moderate ^§^
**Acetminophen**	**−1.88**	**[−2.84; −0.91]**	**−3.82**	**<0.001**	**5**	**0.648**	⊕⊕⊕⊖ Moderate ^§^
Clonidine	−1.51	[−3.37; 0.34]	−1.60	0.109	6	0.554	⊕⊕⊖⊖ Low ^§#^
Ketamine	−0.68	[−1.50; 0.13]	−1.63	0.103	7	0.334	⊕⊕⊖⊖ Low ^§#^
Dexmedetomidine	−0.53	[−1.32; 0.24]	−1.35	0.178	8	0.278	⊕⊕⊖⊖ Low ^§#^
Pregabalin	−0.20	[−1.77; 1.37]	−0.25	0.803	9	0.202	⊕⊕⊖⊖ Low ^§#^
Ketamine + magnesium	−0.17	[−1.54; 1.18]	−0.25	0.799	10	0.182	⊕⊕⊖⊖ Low ^§#^
Placebo	-	-	-	-	11	0.099	
**VAS 30 min**	**τ^2^ = 1.65; τ = 1.28; I^2^ = 88.5% [77.6%; 94.1%]; *p* < 0.001 at Q test**						
**Drug**	**MD**	**95% CI**	**z**	***p* value**	**Rank**	**P score**	**QoE**
Ketamine + clonidine	−2.00	[−4.54; 0.54]	−1.54	0.123	1	0.765	⊕⊕⊖⊖ Low ^§#^
**Dexmedetomidine**	**−1.36**	**[−2.59; −0.12]**	**−2.15**	**0.031**	**2**	**0.639**	⊕⊕⊕⊖ Moderate ^§^
Magnesium	−1.00	[−3.63; 1.63]	−0.74	0.457	3	0.498	⊕⊕⊖⊖ Low ^§^
Ketamine	−0.89	[−2.90; 1.10]	−0.88	0.380	4	0.472	⊕⊕⊖⊖ Low ^§#^
Placebo	-	-	-	-	5	0.124	
**VAS 60 min**	**τ^2^ = 1.3943; τ = 1.18; I^2^ = 88.7% [82.7%; 92.6%]; *p* < 0.001 at Q test**						
**Drug**	**MD**	**95% CI**	**z**	***p* value**	**Rank**	**P score**	**QoE**
**Lidocaine**	**−2.23**	**[−3.61; −0.85]**	**−3.17**	**0.001**	**1**	**0.836**	⊕⊕⊕⊖ Moderate ^§^
Gabapentin	−2.20	[−4.59; 0.19]	−1.80	0.072	2	0.764	⊕⊕⊖⊖ Low ^§#^
**Ketamine**	**−1.31**	**[−2.33; −0.29]**	**−2.53**	**0.011**	**3**	**0.546**	⊕⊕⊕⊖ Moderate ^§^
**Dexmedetomidine**	**−1.24**	**[−2.16; −0.33]**	**−2.67**	**0.007**	**4**	**0.519**	⊕⊕⊕⊖ Moderate ^§^
Ketamine + clonidine	−1.00	[−3.34; 1.34]	−0.83	0.403	5	0.453	⊕⊕⊖⊖ Low ^§#^
Pregabalin	−0.20	[−2.93; 2.53]	−0.14	0.885	6	0.264	⊕⊕⊖⊖ Low ^§#^
Placebo	-	-	-	-	7	0.115	
**VAS 2 h**	**τ^2^ = 0.1162; τ = 0.3408; I^2^ = 50.5% [0.0%; 76.0%]; *p* < 0.001 at Q test**						
**Drug**	**MD**	**95% CI**	**z**	***p* value**	**Rank**	**P score**	**QoE**
**Ibuprofen**	**−2.37**	**[−3.32; −1.41]**	**−4.85**	**<0.001**	**1**	**0.988**	⊕⊕⊕⊖ Moderate ^§^
**Acetaminophen**	**−1.53**	**[−2.48; −0.57]**	**−3.16**	**0.001**	**2**	**0.785**	⊕⊕⊕⊖ Moderate ^§^
**Ketamine**	**−1.03**	**[−1.65; −0.40]**	**−3.23**	**0.001**	**3**	**0.624**	⊕⊕⊕⊖ Moderate ^§^
**Dexmedetomidine**	**−0.87**	**[−1.69; −0.05]**	**−2.10**	**0.035**	**4**	**0.545**	⊕⊕⊕⊖ Moderate ^§^
Gabapentin	−0.60	[−1.80; 0.60]	−0.98	0.328	5	0.418	⊕⊕⊖⊖ Low ^§#^
Lidocaine	−0.28	[−1.25; 0.67]	−0.59	0.557	6	0.278	⊕⊕⊖⊖ Low ^§#^
Magnesium	−0.17	[−1.32; 0.98]	−0.29	0.773	7	0.237	⊕⊕⊖⊖ Low ^§#^
Placebo	-	-	-	-	8	0.121	
**VAS 4 h**	**τ^2^ = 0.2421; τ = 0.4921; I^2^ = 59% [10.4%; 81.2%]; *p* < 0.001 at Q test**						
**Drug**	**MD**	**95% CI**	**z**	***p* value**	**Rank**	**P score**	**QoE**
**Dexmedetomidine**	**−3.10**	**[−5.35; −0.84]**	**−2.70**	**0.006**	**1**	**0.925**	⊕⊕⊕⊖ Moderate ^§^
**Magnesium**	**−2.06**	**[−3.58; −0.53]**	**−2.65**	**0.008**	**2**	**0.783**	⊕⊕⊕⊖ Moderate ^§^
**Ibuprofen**	**−1.63**	**[−2.88; −0.37]**	**−2.54**	**0.011**	**3**	**0.688**	⊕⊕⊕⊖ Moderate ^§^
**Gabapentin**	**−1.19**	**[−2.09; −0.29]**	**−2.59**	**0.009**	**4**	**0.540**	⊕⊕⊕⊖ Moderate ^§^
Ketamine	−1.17	[−2.57; 0.23]	−1.63	0.103	5	0.537	⊕⊕⊖⊖ Low ^§#^
Acetaminophen	−1.10	[−2.34; 0.14]	−1.73	0.083	6	0.496	⊕⊕⊖⊖ Low ^§#^
Ketamine + magnesium	−0.34	[−1.75; 1.07]	−0.47	0.637	7	0.248	⊕⊕⊖⊖ Low ^§#^
Lidocaine	0.04	[−1.53; 1.61]	0.05	0.960	8	0.161	⊕⊕⊖⊖ Low ^§#^
Placebo	-	-	-	-	9	0.118	
**VAS 6 h**	**τ^2^ = 0; τ = 0; I^2^ = 0% [0.0%; 74.6%]; *p* = 0.568 at Q test**						
**Drug**	**MD**	**95% CI**	**z**	***p* value**	**Rank**	**P score**	**QoE ^‡^**
**Lidocaine**	**−2.27**	**[−2.92; −1.63]**	**−6.93**	**<0.001**	**1**	**0.944**	⊕⊕⊕⊖ Moderate ^§^
**Clonidine**	**−1.73**	**[−2.98; −0.48]**	**−2.71**	**0.006**	**2**	**0.814**	⊕⊕⊕⊖ Moderate ^§^
Ketamine + clonidine	−1.00	[−3.04; 1.04]	−0.96	0.338	3	0.571	⊕⊕⊖⊖ Low ^§#^
**Dexmedetomidine**	**−0.44**	**[−0.75; −0.12]**	**−2.74**	**0.006**	**4**	**0.474**	⊕⊕⊕⊖ Moderate ^§^
Ketamine	−0.36	[−0.77; 0.04]	−1.75	0.080	5	0.395	⊕⊕⊖⊖ Low ^§#^
Gabapentin	−0.20	[−0.40; 0.01]	−1.88	0.060	6	0.258	⊕⊕⊖⊖ Low ^§#^
Placebo	-	-	-	-	7	0.041	
**VAS 8 h**	**τ^2^ = 0; τ = 0; I^2^ = 0% [10.4%; 89.6%]; *p* = 1.000 at Q test**						
**Drug**	**MD**	**95% CI**	**z**	***p* value**	**Rank**	**P score**	**QoE ^‡^**
**Ibuprofen**	**−2.50**	**[−2.87; −2.12]**	**−13.16**	**<0.001**	**1**	**0.926**	⊕⊕⊕⊖ Moderate ^§^
**Gabapentin**	**−2.44**	**[−3.17; −1.70]**	**−6.50**	**<0.001**	**2**	**0.896**	⊕⊕⊕⊖ Moderate ^§^
**Acetaminophen**	**−1.80**	**[−2.19; −1.40]**	**−8.96**	**<0.001**	**3**	**0.677**	⊕⊕⊕⊖ Moderate ^§^
Ketamine	−0.34	[−1.17; 0.49]	−0.80	0.423	4	0.426	⊕⊕⊖⊖ Low ^§#^
Placebo	-	-	-	-	5	0.284	
Ketamine + magnesium	−0.00	[−0.84; 0.84]	−0.00	1.000	6	0.274	⊕⊕⊖⊖ Low ^§#^
Magnesium	0.77	[0.23; 1.30]	2.81	0.005	7	0.013	⊕⊕⊖⊖ Low ^§#^
**VAS 12 h**	**τ^2^ = 0.1162; τ = 0.3408; I^2^ = 50.5% [0.0%; 76.0%]; *p* = 0.033 at Q test**						
**Drug**	**MD**	**95% CI**	**z**	***p* value**	**Rank**	**P score**	**QoE**
**Ibuprofen**	**−2.23**	**[−2.87; −1.58]**	**−6.79**	**<0.001**	**1**	**0.964**	⊕⊕⊕⊖ Moderate ^§^
**Lidocaine**	**−1.90**	**[−2.72; −1.08]**	**−4.55**	**<0.001**	**2**	**0.878**	⊕⊕⊕⊖ Moderate ^§^
**Acetaminophen**	**−1.63**	**[−2.27; −0.98]**	**−4.93**	**<0.001**	**3**	**0.801**	⊕⊕⊕⊖ Moderate ^§^
Clonidine	−0.77	[−2.00; 0.44]	−1.25	0.212	4	0.580	⊕⊕⊖⊖ Low ^§#^
**Ketamine**	**−0.43**	**[−0.85; −0.00]**	**−1.97**	**0.048**	**5**	**0.502**	⊕⊕⊕⊖ Moderate ^§^
Gabapentin	−0.30	[−1.11; 0.51]	−0.72	0.468	6	0.411	⊕⊕⊖⊖ Low ^§#^
Dexmedetomidine	−0.19	[−0.71; 0.31]	−0.76	0.450	7	0.354	⊕⊕⊖⊖ Low ^§#^
Ketamine + clonidine	0.00	[−0.76; 0.76]	0.00	1.000	8	0.268	⊕⊕⊖⊖ Low ^§#^
Placebo	-	-	-	-	9	0.231	
Ketamine + magnesium	1.06	[0.20; 1.92]	2.43	0.015	10	0.007	⊕⊕⊖⊖ Low ^§#^
**VAS 24 h**	**τ^2^ = 0.1535; τ = 0.3918; I^2^ = 61.8% [35.4%; 77.4%]; *p* < 0.001 at Q test**						
**Drug**	**MD**	**95% CI**	**z**	***p* value**	**Rank**	**P score**	**QoE**
**Ibuprofen**	**−1.00**	**[−1.57; −0.43]**	**−3.45**	**<0.001**	**1**	**0.919**	⊕⊕⊕⊖ Moderate ^§^
Pregabalin	−0.80	[−1.94; 0.34]	−1.37	0.171	2	0.771	⊕⊕⊖⊖ Low ^§#^
Acetaminophen	−0.45	[−0.91; 0.03]	−1.95	0.051	3	0.649	⊕⊕⊖⊖ Low ^§#^
Clonidine	−0.39	[−1.73; 0.94]	−0.57	0.566	4	0.564	⊕⊕⊖⊖ Low ^§#^
Ketamine	−0.28	[−0.70; 0.12]	−1.36	0.173	5	0.543	⊕⊕⊖⊖ Low ^§#^
Gabapentin	−0.12	[−1.01; 0.77]	−0.26	0.792	6	0.409	⊕⊕⊖⊖ Low ^§#^
Dexmedetomidine	−0.12	[−0.64; 0.39]	−0.46	0.646	7	0.393	⊕⊕⊖⊖ Low ^§#^
Lidocaine	0.10	[−0.93; 1.13]	0.19	0.849	8	0.291	⊕⊕⊖⊖ Low ^§#^
Placebo	-	-	-	-	9	0.278	
Ketamine + magnesium	0.28	[−0.62; 1.19]	0.62	0.536	10	0.179	⊕⊕⊖⊖ Low ^§#^
**VAS 48 h**	**τ^2^ = 0.2421; τ = 0.4921; I^2^ = 59% [10.4%; 81.2%]; *p* = 0.017 at Q test**						
**Drug**	**MD**	**95% CI**	**z**	***p* value**	**Rank**	**P score**	**QoE**
**Dexmedetomidine**	**−0.80**	**[−1.58; −0.02]**	**−2.03**	**0.042**	**1**	**0.771**	⊕⊕⊕⊖ Moderate ^§^
Lidocaine	−0.65	[−1.40; 0.10]	−1.69	0.090	2	0.650	⊕⊕⊖⊖ Low ^§#^
Acetaminophen	−0.60	[−1.90; 0.70]	−0.90	0.366	3	0.575	⊕⊕⊖⊖ Low ^§#^
Ketamine	−0.38	[−0.99; 0.23]	−1.21	0.224	4	0.411	⊕⊕⊖⊖ Low ^§#^
Placebo	-	-	-	-	5	0.090	
**VAS POD7**	**τ^2^ = 0.6176; τ = 0.7859; I^2^ = 67.6% [0.0%; 90.6%]; *p* = 0.045 at Q test**						
**Drug**	**MD**	**95% CI**	**z**	***p* value**	**Rank**	**P score**	**QoE**
Dexmedetomidine	−1.05	[−2.13; 0.02]	−1.91	0.056	1	0.915	⊕⊕⊖⊖ Low ^§#^
Ketamine	0.00	[−1.57; 1.57]	0.00	1.000	2	0.320	⊕⊕⊖⊖ Low ^§#^
Placebo	-	-	-	-	3	0.264	

VAS: visual analogue scale (range from 0 [“no pain”] to 10 [“worst pain possible”]), assessed at various time points; POD7: seventh postoperative day; MD: mean difference; 95% CI: 95% confidence interval; I^2^ measures percentage variation across studies due to heterogeneity; τ^2^: the between-study variance in the random-effects meta-analysis; τ: the standard deviation estimate of effect sizes in the random-effects meta-analysis; Q test: Cochran’s Q test assesses heterogeneity among the study results; z: the Z-score measures how many standard deviations a data point is from the mean; rank: the effectiveness-based order of treatments; QoE: quality of evidence; ^§^: downgraded one level for inconsistency (such as heterogeneity of estimates of effects across trials) [11]. ^#^: downgraded one level for imprecision (for example, 95% confidence intervals are wide and include or are close to null effect) [11]. Moderate QoE: the authors are moderately confident in the effect estimate. The true effect is likely to be close to the estimate of the effect, but there is a possibility that it is substantially different. Low QoE: the authors’ confidence in the effect estimate is limited. The true effect may be substantially different from the estimate of the effect [11]. **^‡^**: the heterogeneity was considered uncertain. This uncertainty arises from a broad confidence interval for I^2^, suggesting the possibility of undetected heterogeneity. This concern remains despite the reported absence of between-study variance (τ^2^ = 0), no variation in effect estimates (τ = 0), and a reported I^2^ value of 0%.

#### 3.4.2. Use of Rescue Analgesics

The use of additional analgesics was evaluated in 10 studies [16,17,22,23,27,28,29,30,39,44], with ibuprofen significantly reducing the need for additional analgesics within 24 h post-surgery (OR 0.34). Other treatments showed no significant reduction compared to a placebo/no intervention or a comparator at any other time point (Table 2). The SUCRA rankings are detailed in Table 2 and visualized as a graph in Figure S2. The quality of evidence (QoE) for the measured outcomes is detailed in Table 2. The study analysis for rescue therapy at various postoperative time points yielded the following QoE assessments: at the Post Anesthesia Care Unit (PACU), 25% of studies contributed to a moderate QoE and 75% to a low QoE; within 6 h, all studies (100%) contributed to a low QoE; within 24 h, all studies (100%) contributed to a moderate QoE; and within 48 h, all studies (100%) contributed to a low QoE.

**Table 2:** Effects of intravenous non-opioid agents and adjuvants on postoperative rescue analgesia, PONV, and recovery quality.

Rescue Therapy at PACU	τ^2^ = NA; τ = NA; I^2^ NA						
Drug	OR	95% CI	z	*p* Value	Rank	P Score	QoE *^‡^*
Placebo	-	-	-	-	1	0.784	-
Pregabalin	1.50	[0.53; 4.17]	0.78	0.437	2	0.516	⊕⊕⊖⊖ Low ^§#^
**Ibuprofen**	**2.29**	**[0.18; 27.80]**	**0.65**	**0.024**	**2**	**0.449**	⊕⊕⊕⊖ Moderate ^§^
Acetaminophen	3.04	[0.30; 30.12]	0.95	0.340	1	0.248	⊕⊕⊖⊖ Low ^§#^
**Rescue therapy within 6 h**	**τ^2^ = NA; τ = NA; I^2^ NA**						
**Drug**	**OR**	**95% CI**	**z**	***p* value**	**Rank**	**P score**	**QoE** ***^‡^***
Gabapentin	0.34	[0.11; 1.05]	−1.87	0.062	-	-	⊕⊕⊖⊖ Low ^§#^
Placebo	-	-	-	-	-	-	-
**Rescue therapy within 24 h**	**τ^2^ = 0; τ = 0; I^2^ 0%; *p* = 1.000 at Q test**						
**Drug**	**OR**	**95% CI**	**z**	***p* value**	**Rank**	**P score**	**QoE**
Ibuprofen	0.34	[0.01; 8.58]	−0.65	0.516	1	0.711	⊕⊕⊕⊖ Moderate ^#^
Lidocaine	0.60	[0.22; 1.64]	−0.99	0.323	2	0.673	⊕⊕⊕⊖ Moderate ^#^
Pregabalin	0.55	[0.12; 2.56]	−0.75	0.451	3	0.670	⊕⊕⊕⊖ Moderate ^#^
Ketamine + magnesium	0.75	[0.21; 2.61]	−0.45	0.651	4	0.575	⊕⊕⊕⊖ Moderate ^#^
Placebo	-	-	-	-	5	0.419	-
Ketamine	1.41	[0.38; 5.26]	0.52	0.603	6	0.264	⊕⊕⊕⊖ Moderate ^#^
Acetaminophen	1.63	[0.63; 4.20]	1.02	0.309	7	0.185	⊕⊕⊕⊖ Moderate ^#^
**Rescue therapy within 48 h**	**τ^2^ = 0.801; τ = 0.895; I^2^ = 51.2% [0.0%; 85.9%]; *p* = 0.128 at Q test**						
**Drug**	**OR**	**95% CI**	**z**	***p* value**	**Rank**	**P score**	**QoE**
Ketamine	0.15	[0.01; 1.68]	−1.53	0.126	1	0.879	⊕⊕⊖⊖ Low ^§#^
Lidocaine	0.49	[0.02; 9.81]	−0.46	0.643	2	0.602	⊕⊕⊖⊖ Low ^§#^
Placebo	-	-	-	-	3	0.436	-
Dexmedetomidine	2.82	[0.68; 11.65]	1.44	0.150	4	0.082	⊕⊕⊖⊖ Low ^§#^
**PONV**	**τ^2^ = 0.255; τ = 0.505; I^2^ = 44.1% [11.8%; 64.5%; *p* = 0.008 at Q test**						
**Drug**	**OR**	**95% CI**	**z**	***p* value**	**Rank**	**P score**	**QoE**
**Pregabalin + dexmedetomidine**	**0.06**	**[0.00; 0.72]**	**−2.23**	**0.025**	**1**	**0.918**	⊕⊕⊕⊖ Moderate ^§^
**Clonidine**	**0.16**	**[0.03; 0.79]**	**−2.26**	**0.024**	**2**	**0.822**	⊕⊕⊕⊖ Moderate ^§^
**Dexmedetomidine**	**0.30**	**[0.18; 0.50]**	**−4.53**	**<0.001**	**3**	**0.707**	⊕⊕⊕⊖ Moderate ^§^
**Ibuprofen**	**0.32**	**[0.11; 0.91]**	**−2.14**	**0.032**	**4**	**0.662**	⊕⊕⊕⊖ Moderate ^§^
**Gabapentin**	**0.33**	**[0.12; 0.91]**	**−2.13**	**0.033**	**5**	**0.648**	⊕⊕⊕⊖ Moderate ^§^
Magnesium	0.39	[0.13; 1.10]	−1.70	0.077	6	0.582	⊕⊕⊖⊖ Low ^§#^
Lidocaine	0.63	[0.63; 1.07]	−1.68	0.093	7	0.375	⊕⊕⊖⊖ Low ^§#^
Ketamine	0.77	[0.42; 1.43]	−0.80	0.421	8	0.271	⊕⊕⊖⊖ Low ^§#^
Acetaminophen	0.82	[0.36; 1.85]	−0.47	0.641	9	0.242	⊕⊕⊖⊖ Low ^§#^
Pregabalin	1.10	[0.40; 2.99]	0.20	0.845	10	0.142	⊕⊕⊖⊖ Low ^§#^
Placebo	-	-	-	-	11	0.125	-
**QoR40 POD1**	**τ^2^ = 0; τ = 0; I^2^ = 0%; *p* = 0.539 at Q test**						
**Drug**	**MD**	**95% CI**	**z**	***p* value**	**Rank**	**P score**	**QoE**
Placebo	-	-	-	-	1	0.905	-
Pregabalin	1.60	[−3.76; 6.96]	0.58	0.559	2	0.743	⊕⊕⊕⊖ Moderate ^#^
**Ketamine**	**8.66**	**[2.41; 14.90]**	**2.72**	**0.006**	**3**	**0.229**	⊕⊕⊕⊕ High
**Lidocaine**	**9.88**	**[7.18; 12.59]**	**7.16**	**<0.001**	**4**	**1.121**	⊕⊕⊕⊕ High
**QoR40 POD 3**	**τ^2^ = 0; τ = 0; I^2^ = 0%; *p* = 1.000 at Q test**						
**Drug**	**MD**	**95% CI**	**z**	***p* value**	**Rank**	**P score**	**QoE**
Placebo	-	-	-	-	1	1.000	-
**Lidocaine**	**33.00**	**[31.24; 34.75]**	**36.96**	**<0.001**	**2**	**0.500**	⊕⊕⊕⊕ High
**Dexmedetomidine**	**46.00**	**[44.47; 47.52]**	**59**	**0**	**3**	**0.000**	⊕⊕⊕⊕ High

PACU: post-anesthesia care unit; PONV: postoperative nausea and vomiting; QoR-40: quality of recovery-40, a questionnaire; POD1 and POD3: first and third postoperative days; MD: mean difference; 95% CI: 95% confidence interval; OR: odds ratio; I^2^ measures percentage variation across studies due to heterogeneity; τ^2^: between-study variance in the random-effects meta-analysis; τ: the standard deviation estimate of effect sizes in the random-effects meta-analysis; Q test: Cochran’s Q test assesses heterogeneity among the study results; z: the Z-score measures how many standard deviations a data point is from the mean; rank: the effectiveness-based order of treatments; QoE: quality of evidence. ^§^: downgraded one level for Inconsistency (such as heterogeneity of estimates of effects across trials) [11]. ^#^: downgraded one level for imprecision (for example, 95% confidence intervals are wide and include or are close to null effect) [11]. High QoE: the authors are very confident that the true effect lies close to that of the estimate of the effect. Moderate QoE: the authors are moderately confident in the effect estimate. The true effect is likely to be close to the estimate of the effect, but there is a possibility that it is substantially different. Low QoE: the authors’ confidence in the effect estimate is limited. The true effect may be substantially different from the estimate of the effect [11]. ***^‡^***: the absence of reported heterogeneity (τ^2^, τ, and I^2^ values not available) should not be interpreted as definitive evidence of no variability among the study effects. Accordingly, caution has been exercised in the assessment, acknowledging that the QoE may be impacted by potential, yet undetected, heterogeneity.

#### 3.4.3. PONV

PONV was evaluated in 29 studies [15,16,17,19,20,21,22,23,24,26,27,28,30,31,32,33,34,35,37,38,40,41,44,45,46,47,48,49,51,52]. Pregabalin plus dexmedetomidine (OR 0.06) [24], clonidine (OR 0.16) [22], dexmedetomidine (OR 0.30) [15,17,22,26,31,47,49], ibuprofen (OR 0.32) [28,33], or gabapentin (OR 0.33) [23,32,35] resulted in a statistically significant reduction in PONV incidence when compared to a placebo/no intervention or a comparator. The SUCRA rankings are detailed in Table 2 and visualized as a graph in Figure S3. The QoE for the measured outcome of PONV is detailed in Table 2. In the study analysis for PONV, 45.2% of studies contributed to a moderate QoE and 54.8% to a low QoE.

#### 3.4.4. Quality of Recovery-40 (QoR-40)

The QoR-40 was evaluated in five studies [21,26,30,46,52]. Compared to a placebo/no intervention or a comparator, ketamine (MD 8.66) [52] and lidocaine (MD 9.88) [21,46] significantly increased the quality of postoperative recovery in patients, as assessed on the first postoperative day. Compared to a placebo, lidocaine (MD 33) [26] and dexmedetomidine (MD 46) [26] significantly increased the quality of postoperative recovery in patients, as assessed on the third postoperative day (Table 2). The SUCRA rankings are detailed in Table 2. The QoE for the measured outcomes is detailed in Table 2. In the analysis, 75% of studies contributed to a high QoE and 25% to a moderate QoE for the QoR40 score 1 day post-surgery. All studies (100%) contributed to a high QoE for the QoR40 score 3 days post-surgery.

## 4. Discussion

Our systematic review reveals that intravenous non-opioid analgesics and adjuvants, when used within a multimodal anesthesia framework, significantly enhance postoperative pain management for obese patients. NSAIDs, acetaminophen (or paracetamol), lidocaine, α-2 agonists, ketamine, and oral gabapentinoids are effective in reducing early postoperative pain. NSAIDs, particularly ibuprofen, alongside acetaminophen, ketamine, and lidocaine, also offer benefits during the later stages of postoperative recovery. While all the non-opioid analgesics and adjuvants assessed provide some degree of pain relief, NSAIDs are distinguished by their effectiveness at various postoperative times and in reducing the need for additional analgesics, especially at the end of surgery. A-2 agonists, when used alone or in combination with oral gabapentinoids, significantly lower the risk of PONV. However, the evidence supporting the effectiveness of these agents in managing pain beyond the first two postoperative days is limited. Nonetheless, ketamine, lidocaine, and α-2 agonists, such as dexmedetomidine, are promising in improving the quality of postoperative care and recovery.

Various meta-analyses have shown the effectiveness of drugs like NSAIDs [53], acetaminophen (or paracetamol) [54], ketamine [55], dexmedetomidine [56,57], clonidine [58], lidocaine [59], and magnesium [60,61], though not conclusively in obese patients [62], and preoperative oral gabapentinoids [63] in providing opioid-sparing analgesia. These medications have been effective in improving postoperative pain relief [53,54,55,56,57,58,59,60,61,62,63], reducing PONV [53,55,56,57,58,63], and enhancing recovery quality in obese patients undergoing surgery [53,54,55,56,57,58,59,60,61,62,63].

Our analysis indicates that NSAIDs, particularly ibuprofen, effectively manage pain during most of the postoperative period, underscoring that NSAIDs are very good at controlling pain after surgery [53]. NSAIDs are increasingly used over acetaminophen and opioids due to their better pain relief and anti-inflammatory effects [53]. Recent studies confirm NSAIDs are safe and well tolerated in bariatric surgery, effectively easing postoperative pain with minimal safety issues [53]. A further analysis revealed that ibuprofen, with a 2 h half-life, showed a risk ratio (RR) of 1.44 for achieving at least 50% of maximum pain relief over 4 and 6 h, compared to a placebo [64]. Similarly, ketorolac, another NSAID with a 4 to 6 h half-life, resulted in an RR of 2.81 for significant pain relief over 4 h and 3.26 over 6 h, compared to a placebo [65]. Nonetheless, there was no significant difference in pain relief when comparing ibuprofen with acetaminophen [64] or ketorolac with other NSAIDs [65] for the durations observed [64,65].

Previous meta-analyses have found no significant difference in pain scores at 12 and 24 h postoperatively between intravenous acetaminophen (or paracetamol) and other comparators in adults undergoing abdominal surgery [66]. Our findings confirm that acetaminophen (or paracetamol) provides a consistent analgesic effect, aligning with the existing literature on its effectiveness in surgical patients suffering from obesity, particularly after bariatric surgery, in reducing pain scores 24 h postoperatively [54].

An earlier meta-analysis has demonstrated that administering intravenous ketamine during bariatric surgery significantly reduces pain scores immediately after the procedure and provides modest benefits for up to 24 h postoperatively [55]. Our study confirms that ketamine significantly lowers postoperative pain scores within the first 12 h following surgery, with a reduced effect thereafter. In non-bariatric surgeries, intravenous S-ketamine during general anesthesia has been shown through a meta-analysis to enhance resting pain scores at 4, 12, and 24 h after surgery compared to a placebo, although these advantages do not extend beyond the first day [67].

Based on our meta-analysis, dexmedetomidine has been shown to provide pain relief during the early postoperative period, peaking in analgesic effectiveness 4 h after surgery and significantly diminishing afterward. This indicates a strong but short-lived analgesic effect. Our findings, aligning with the terminal half-life of 2–3 h, corroborate previous evidence on the analgesic benefits of intraoperative dexmedetomidine infusions. These benefits have been observed to extend into the PACU period, with a reduction in pain scores of nearly 25% on a 10-point scale [56,57], and persisting six hours postoperatively, with a reduction in pain scores of nearly 20% [57].

A previous meta-analysis has revealed that clonidine did not reduce resting pain scores in the majority of studies involving adult surgical patients [58]. Our study finds that when used for post-operative pain management, clonidine demonstrates a significant analgesic effect 6 h after surgery, but not beyond this time frame. This observation is consistent with the pharmacokinetic properties of clonidine, which has a half-life of approximately 8–12 h [58].

A meta-analysis has highlighted the advantages of intraoperative intravenous lidocaine, notably in decreasing the time to first opioid requirement and improving recovery quality, although it did not significantly impact postoperative pain scores at 24 h [59]. Our study finds that lidocaine is highly effective in the early postoperative period, specifically within the 1st hour after surgery, and again later, between 6 to 12 h post-surgery. However, this effect significantly diminishes by the 24 h and 48 h marks, suggesting an immediate but transient analgesic impact.

The literature has underscored the positive effects of systemic magnesium in diminishing postoperative pain among general surgical patients [60,61], with observed reductions in both early and late resting pain, as well as late movement-related pain. However, the evidence in bariatric surgery settings appears less conclusive, possibly due to the scarcity of data [66]. In our study, despite a general lack of data across most time points, magnesium shows a significant peak in effectiveness 4 h post-surgery, indicating a potential delayed analgesic effect or a time-specific peak in efficacy.

Our meta-analysis confirms that oral gabapentinoids significantly reduce pain scores within the first 4 h after surgery, with the benefit of pain relief extending over the following 4 h [63]. This is supported by our analysis, which demonstrates a benefit within the first 8 h postoperatively, showing strong initial efficacy that decreases over time. This trend indicates a diminishing effect of pain relief as time progresses, likely due to the peak plasma concentration occurring 1–3 h after oral intake and the elimination half-life of 5–9 h [68]. Our findings are in line with previous research, underscoring the effectiveness of oral gabapentinoids in managing post-surgical pain up to 12 h [63].

The observed benefit of pain relief at various time points, provided by intravenous non-opioid analgesics and adjuvants compared to a placebo/no intervention or comparators, may elucidate the noted impact on the reduction in analgesic rescue treatment needed. The recognized efficacy of NSAIDs in managing postoperative pain in bariatric surgery patients, as highlighted in the literature [53], underscores their significant role in reducing the necessity for rescue therapy [53]. This is not surprising given the well-documented efficacy of NSAIDs in this context. While the current meta-analysis did not specifically investigate the opioid-sparing effect of intravenous non-opioid analgesics and adjuvants—that is, their capacity to reduce systemic opioid consumption—the literature extensively documents the benefits of all investigated intravenous non-opioid analgesics and adjuvants in decreasing analgesic consumption [53,54,55,56,57,58,59,60,61,62,63]. This suggests a broader implication for their role in enhancing postoperative pain management strategies.

PONV continues to be a significant adverse event, with an estimated prevalence of 21% in patients with obesity undergoing surgery [53]. Our analysis suggests that NSAIDs, α-2 agonists, and lidocaine significantly reduce PONV, with α-2 agonists showing particularly high efficacy. These agents mitigate PONV through both direct and indirect pathways, capitalizing on their sedative and analgesic effects [56,57,58]. Ibuprofen and oral gabapentinoids, notably gabapentin, display moderate effectiveness, likely due to their analgesic properties that indirectly reduce PONV by alleviating post-operative pain—an independent risk factor for PONV after bariatric surgery, along with female sex (OR = 1.64) and postoperative opioid use (OR = 2.22) [69]. Magnesium, lidocaine, ketamine, and acetaminophen (or paracetamol) are not found to have a significant impact, indicating they may not be as effective in preventing PONV, though a beneficial effect compared to a placebo cannot be entirely ruled out for some of these agents according to other analyses [59].

Despite the paucity of data, the results of our meta-analysis are encouraging for the use of intravenous non-opioid analgesics and adjuvants over a placebo/no intervention or comparators in enhancing recovery quality. Among these, ketamine, α-2 agonists (especially dexmedetomidine), and lidocaine are associated with significant improvements in recovery quality. The adoption of multimodal analgesia, aimed at enhancing postoperative pain relief as part of opioid-sparing strategies, should be prioritized to improve recovery quality within enhanced recovery programs [1,70], as strongly recommended in the perioperative care of patients with obesity [5,6].

The use of multiple intravenous non-opioid agents and adjuvants within the framework of multimodal analgesia has been minimally studied. However, the strategic combination of various intravenous non-opioid agents and adjuvants has been shown to significantly alleviate postoperative pain [18] and enhance postoperative recovery by reducing the risk of adverse events, such as PONV [24]. This suggests that a synergistic multimodal regimen yields better outcomes than employing a single treatment modality [18,24].

### The Strengths and Limitations of the Study

The research highlights the advantages of conducting a comprehensive literature review, which significantly lowers the risk of missing important studies and ensures a strong data set for analysis. The research utilizes a sophisticated analytical technique, network meta-analysis, which provides wider comparative insights compared to conventional methods that examine treatments separately. Additionally, this study meticulously selects trials focusing solely on RCTs involving adults with obesity undergoing surgery. This ensures that the findings are highly relevant and directly applicable to this specific patient group. This focused approach enhances the clarity and pertinence of our results, laying a solid groundwork for our conclusions. Finally, incorporating the GRADE assessment into our analysis stands as a key strength of this study [11]. This systematic approach provides a clear evaluation of evidence certainty, enriching our findings’ reliability and applicability. By rigorously applying the GRADE framework, this study offers readers a transparent insight into the confidence level of conclusions, aligning this work with the best practices in evidence-based healthcare.

However, our study recognizes a number of limitations. The majority of trials we reviewed were conducted in single-center settings, which may limit the generalizability of our findings across varied clinical contexts. Additionally, there was a disparity in the evidence available for each intervention, with certain treatments being underrepresented. Moreover, not all treatments considered in our study were evaluated at every time point analyzed, which could have impacted the reliability and comprehensiveness of our overall analysis. Additionally, considerable heterogeneity was observed across the trials, potentially influencing the comparative effectiveness of the assessments. The limitations of the study include inconsistencies within the evidence network, particularly in the lidocaine comparisons shown in the 60 min VAS heatmap. These inconsistencies suggest possible methodological heterogeneity among the included studies, which could affect the reliability of the specific treatment effect estimates. The analysis overlooked several factors that may influence pain sensitivity, including psychological factors and pre-surgery chronic pain management, potentially diminishing the precision of our findings. The study exclusively considered the intravenous non-opioid analgesics and adjuvants that were included in the presentation of the rationale for multimodal general anesthesia by Brown EN et al. [3], overlooking other utilized drugs such as COX-2 inhibitors, tramadol, nefopam, metamizol, and corticosteroids. Including these interventions in future research could broaden our understanding of pain management strategies. Additionally, the impact of drug dosage was not investigated. In obesity, due to physiological and anthropometric changes that can significantly alter the pharmacokinetics of many drugs, drug dosing must be carefully tailored [1]. While non-opioid analgesics and acetaminophen usually do not need weight adjustments and guidelines for drugs like gabapentinoids and magnesium are unclear, drugs such as ketamine, α-2 agonists, and lidocaine require weight-based dosing for efficacy, underlining the need for precise dosing strategies to ensure safety and effectiveness [1,71]. The analysis of chronic pain data is limited by the absence of information regarding the role of intravenous non-opioid analgesics and adjuvants in multimodal anesthesia for chronic pain treatment of patients with obesity undergoing surgery. This highlights a significant research need to better understand chronic pain management and its impact on outcomes. Pursuing this research direction could yield invaluable insights into optimizing pain management strategies for postoperative care. Addressing these limitations in future studies has the potential to significantly improve the understanding of the effectiveness of pain management and the impact of various interventions in postoperative scenarios.

## 5. Conclusions

This study underscores the critical role of intravenous non-opioid analgesics and adjuvants within a multimodal anesthesia framework for optimizing postoperative pain management in obese patients. Specifically, NSAIDs (notably ibuprofen), acetaminophen (or paracetamol), lidocaine, α-2 agonists, ketamine, and oral gabapentinoids emerge as key agents for alleviating early postoperative pain, with ibuprofen and acetaminophen, along with ketamine and lidocaine, extending their benefits into the later recovery phases. Notably, ibuprofen stands out for its extended pain control efficacy, serving as a pivotal component of prolonged pain management strategies. Furthermore, dexmedetomidine’s effectiveness in the post-24 h period positions it as a valuable asset for sustained pain relief when other analgesics’ effects wane. By integrating these agents at various stages, a multimodal approach not only mitigates adverse outcomes like PONV and the need for rescue analgesia, but also presents a comprehensive, patient-centric solution to postoperative pain, adapting to its evolving nature throughout the recovery process.

## Data Availability

The datasets used and analyzed during the current study are available from the corresponding author upon reasonable request.

## Supplementary Materials

The following supporting information can be downloaded at: https://www.mdpi.com/article/10.3390/jcm13072100/s1, PRISMA 2020 checklist; Supplementary Materials Content (SMC)1: Search strategy; SMC2: Study characteristics; SMC3: “Traffic light” plots of RoB 2 assessment for the included RCTs; SMC4: Reasons for the risk of bias judgments; SMC5: Network graphs; SMC6: Forest plots; SMC7: SUCRA rankings graphs (Figures S1–S3); SMC8: Funnel plots; SMC9: Rankograms; SMC10: Heat maps from the separate indirect from direct evidence (SIDE) analysis.
