# Supplementary material for: Non-Opioid Analgesics and Adjuvants after Surgery in Adults with Obesity: Systematic Review with Network Meta-Analysis of Randomized Controlled Trials
Source: J Clin Med. 2024 Apr 3;13(7):2100. doi: 10.3390/jcm13072100 (PMC11012569; doi:10.3390/jcm13072100)
Supplement: Supplementary file 1 [file jcm-13-02100-s001.zip › SMC_JCM_R1/SMC9. Rankograms. 04.03.24.pdf]

Rankograms Generated From Network Meta-Analysis for Different Time Points and Various Variables Considered

Below, a series of rankograms derived from the network meta-analysis, which has been methodically conducted across different time points and for a selection of variables including Postoperative Nausea and Vomiting (PONV), Use of Rescue Analgesics, and Quality of Recovery-40 (QoR-40), are presented. Rankograms are statistical illustrations that convey the probability distribution of each treatment's rank in terms of effectiveness for a given outcome.

Each rankogram is constructed with precision, mapping the treatments on the x-axis according to their potential effectiveness ranks—ranging from 1, denoting the most effective, to higher values (>1), the least effective. The y-axis corresponds to the probability of each treatment achieving these ranks based on the aggregated data from the meta-analysis. The height of the bars in the rankogram represents this probability, with the tallest bar indicating the most probable ranking for a treatment. Rankograms require a comparison of three or more treatment options to create a meaningful probability distribution for each treatment's rank in terms of effectiveness. With only two substances, the analysis would simply reflect a direct comparison without the probabilistic ranking that rankograms are designed to provide, and therefore a rankogram cannot be provided.

SUPPLEMENTARY MATERIAL CONTENT (SMC) 9 — Rankogram

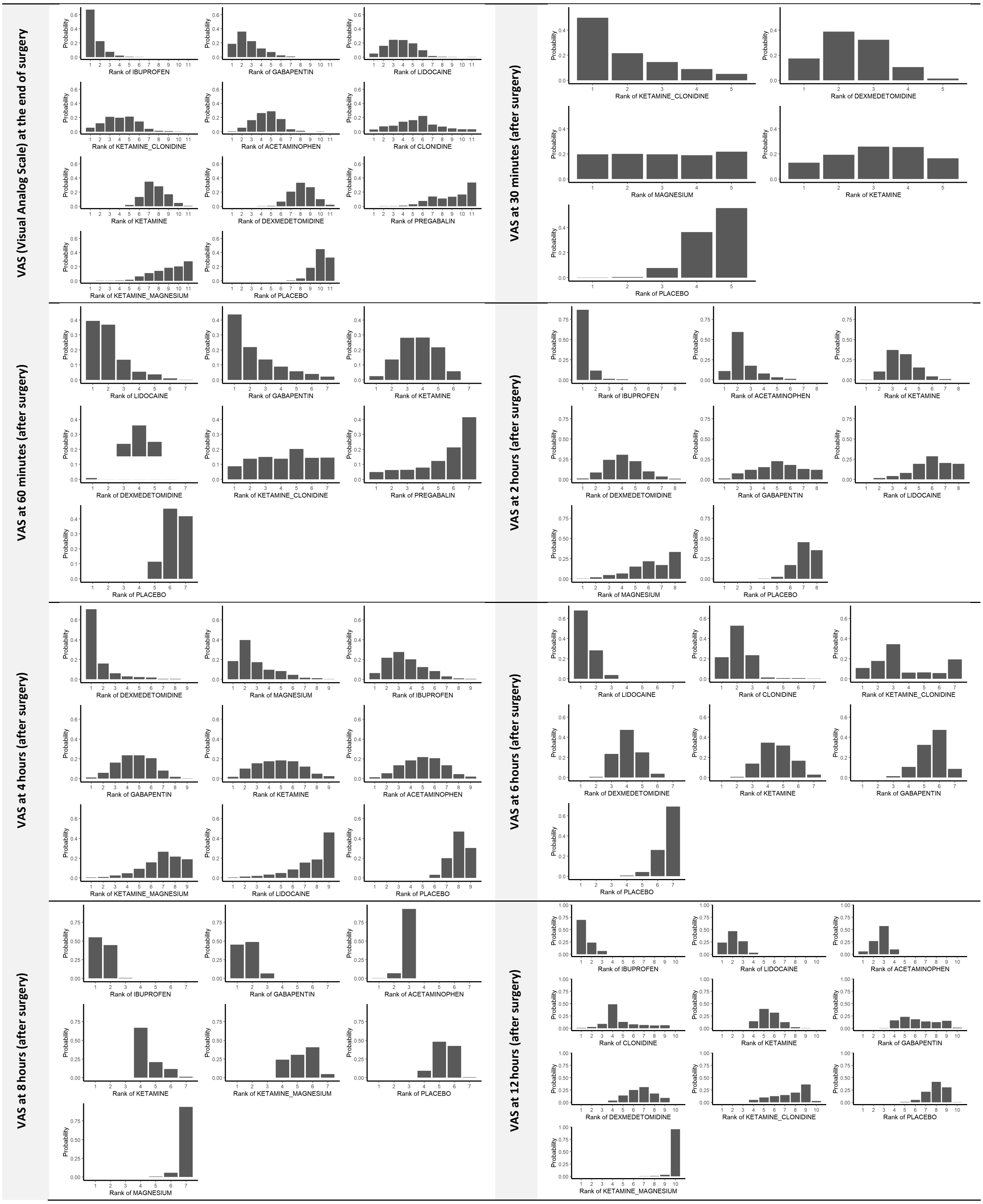

SUPPLEMENTARY MATERIAL CONTENT (SMC) 9 — Rankogram

VAS at 24 hours (after surgery)

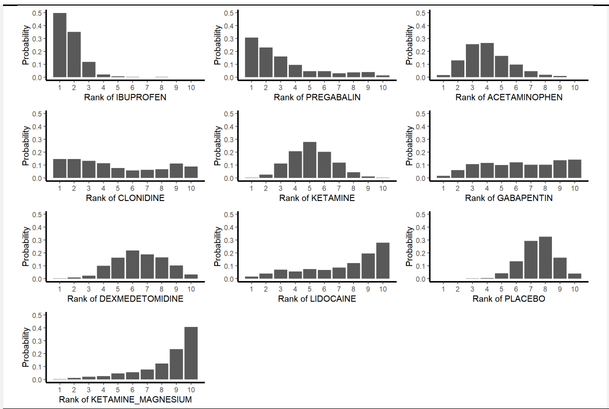

VAS at 48 hours (after surgery)

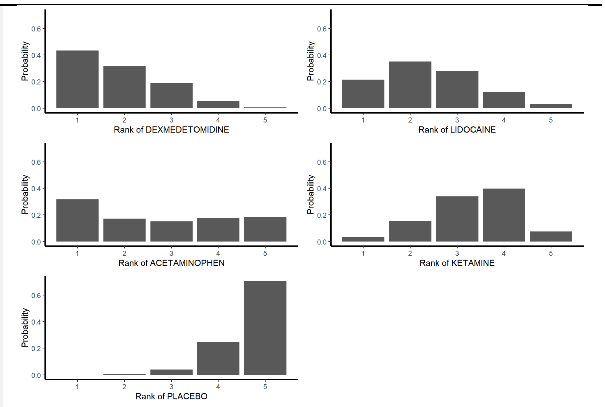

VAS at 7 days post-surgery

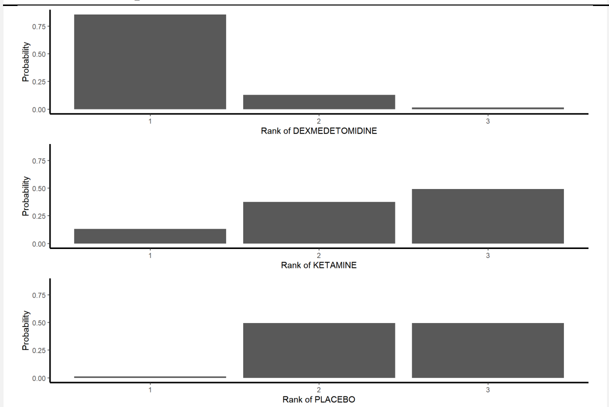

PONV

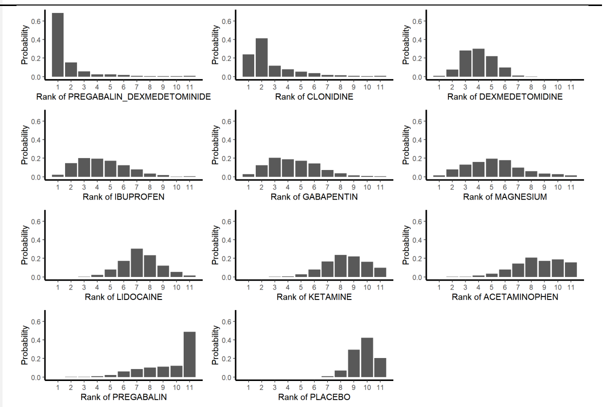

Rescue therapy during PACU stay

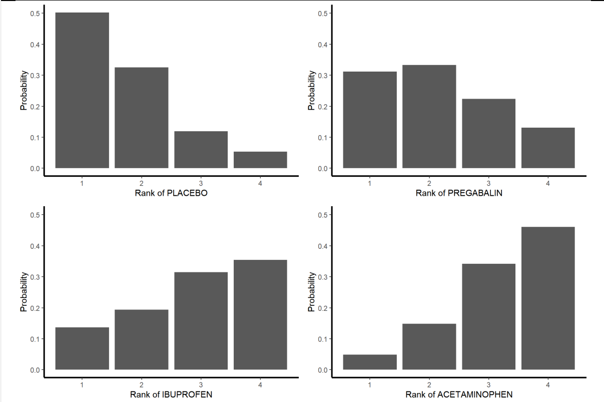

Rescue therapy within 6 hours (after surgery)

Not available

Rescue therapy within 24 hours (after surgery)

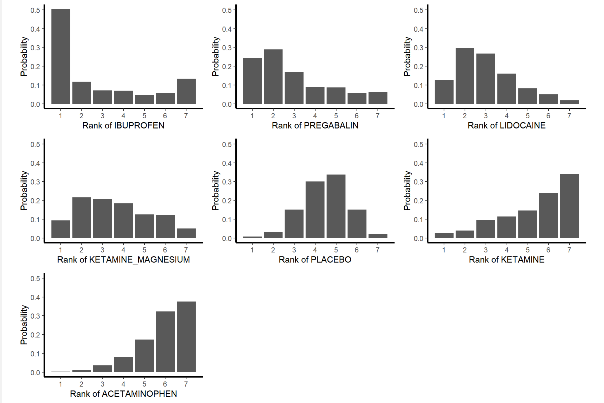

Rescue therapy within 48 hours (after surgery)

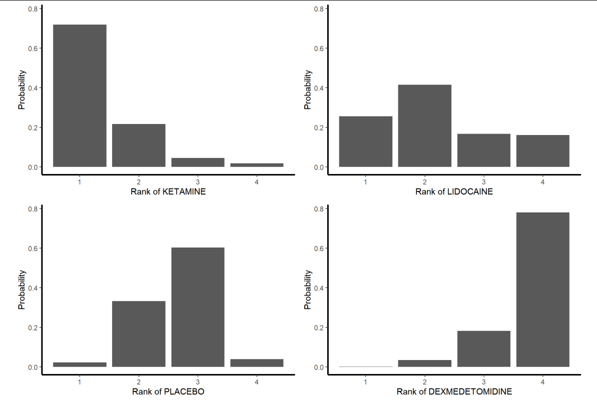

SUPPLEMENTARY MATERIAL CONTENT (SMC) 9 Rankogram

QoR40 on Postoperative Day 1

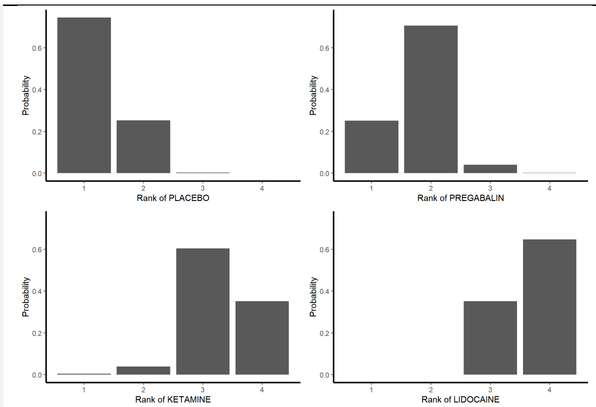

QoR40 on Postoperative Day 3

Not available
