# Supplementary material for: Non-Opioid Analgesics and Adjuvants after Surgery in Adults with Obesity: Systematic Review with Network Meta-Analysis of Randomized Controlled Trials
Source: J Clin Med. 2024 Apr 3;13(7):2100. doi: 10.3390/jcm13072100 (PMC11012569; doi:10.3390/jcm13072100)
Supplement: Supplementary file 1 [file jcm-13-02100-s001.zip › SMC_JCM_R1/SMC1. Search strategy. 02.03.24.pdf]

Search strategy

A comprehensive literature search was conducted to identify relevant studies for inclusion in this systematic review and network meta-analysis. The databases queried included PubMed, Scopus, Web of Science, CINAHL, and EMBASE, with the search being carried out up to September 28, 2023.

**PubMed**	Search: **((obesity, abdominal[MeSH Terms]) OR (obesity, morbid[MeSH Terms]) OR (bariatric surgery[MeSH Terms]) OR (obese)) AND ((magnesium sulfate[MeSH Terms]) OR (lidocaine[MeSH Terms]) OR (Adrenergic alpha-2 Receptor Agonists[MeSH Terms]) OR (ketamine[MeSH Terms]) OR (acetaminophen[MeSH Terms]) OR (Anti-Inflammatory Agents, Non-Steroidal[MeSH Terms]) OR (Analgesics, Non-Narcotic[MeSH Terms]) OR (Gabapentin[MeSH Terms]) OR (Pregabalin[MeSH Terms]) OR (gamma-Aminobutyric Acid[MeSH Terms]) OR (dexmedetomidine[MeSH Terms]) OR (clonidine[MeSH Terms]) OR (ketorolac[MeSH Terms]))** Sort by: **Most Recent** ("obesity, abdominal"[MeSH Terms] OR "obesity, morbid"[MeSH Terms] OR "bariatric surgery"[MeSH Terms] OR ("obeses"[All Fields] OR "obesity"[MeSH Terms] OR "obesity"[All Fields] OR "obese"[All Fields] OR "obesities"[All Fields] OR "obesity s"[All Fields])) AND ("magnesium sulfate"[MeSH Terms] OR "lidocaine"[MeSH Terms] OR "adrenergic alpha 2 receptor agonists"[MeSH Terms] OR "ketamine"[MeSH Terms] OR "acetaminophen"[MeSH Terms] OR "anti inflammatory agents, non steroidal"[MeSH Terms] OR "analgesics, non narcotic"[MeSH Terms] OR "gabapentin"[MeSH Terms] OR "pregabalin"[MeSH Terms] OR "gamma aminobutyric acid"[MeSH Terms] OR "dexmedetomidine"[MeSH Terms] OR "clonidine"[MeSH Terms] OR "ketorolac"[MeSH Terms])

SUPPLEMENTARY MATERIAL CONTENT (SMC) 1	SEARCH STRATEGY
**Scopus**	obesity AND<br>dexmedetomidine OR clonidine OR magnesium AND sulphate OR ketamine OR gabapentin OR pregabalin OR acetaminophen OR ketorolac OR lidocaine)
**Web of Science**	#2 AND #1<br>2 (((((((ALL=(lidocaine)) OR ALL=(dexmedetomidine)) OR ALL=(clonidine)) OR ALL=(ketamine)) OR ALL=(acetaminophen)) OR ALL=(ketorolac)) OR ALL=(gabapentin)) OR ALL=(pregabalin)<br>1 ALL=(obesity)
**Cinhal**	obesity AND (ketorolac OR ketamine OR "magnesium sulphate" OR clonidine OR pregabaline OR gabapentin OR lidocaine OR dexmedetomidine OR NSAID OR acetaminophen OR "non steroidal anti inflammatory" OR ketorolac)
**Embase**	1 (Obesity, Abdominal or Obesity, Morbid or Bariatric Surgery).sh. or obese.af.<br>2 (Magnesium Sulfate or Lidocaine or Adrenergic alpha-2 Receptor Agonists or Ketamine or Acetaminophen or Anti-Inflammatory Agents, Non-Steroidal or Analgesics, Non-Narcotic or Gabapentin or Pregabalin or gamma-Aminobutyric Acid or Dexmedetomidine or Clonidine or Ketorolac).sh.<br>3 1 and 2
