# Supplementary material for: Non-Opioid Analgesics and Adjuvants after Surgery in Adults with Obesity: Systematic Review with Network Meta-Analysis of Randomized Controlled Trials
Source: J Clin Med. 2024 Apr 3;13(7):2100. doi: 10.3390/jcm13072100 (PMC11012569; doi:10.3390/jcm13072100)
Supplement: Supplementary file 1 [file jcm-13-02100-s001.zip › SMC_JCM_R1/SMC7. SUCRA ranking of the results in this meta-analysis. 04.03.24.pdf]

Fig.S1. SUCRA ranking for impact of intravenous non-opioid analgesics and adjuvants on pain relief at different time points

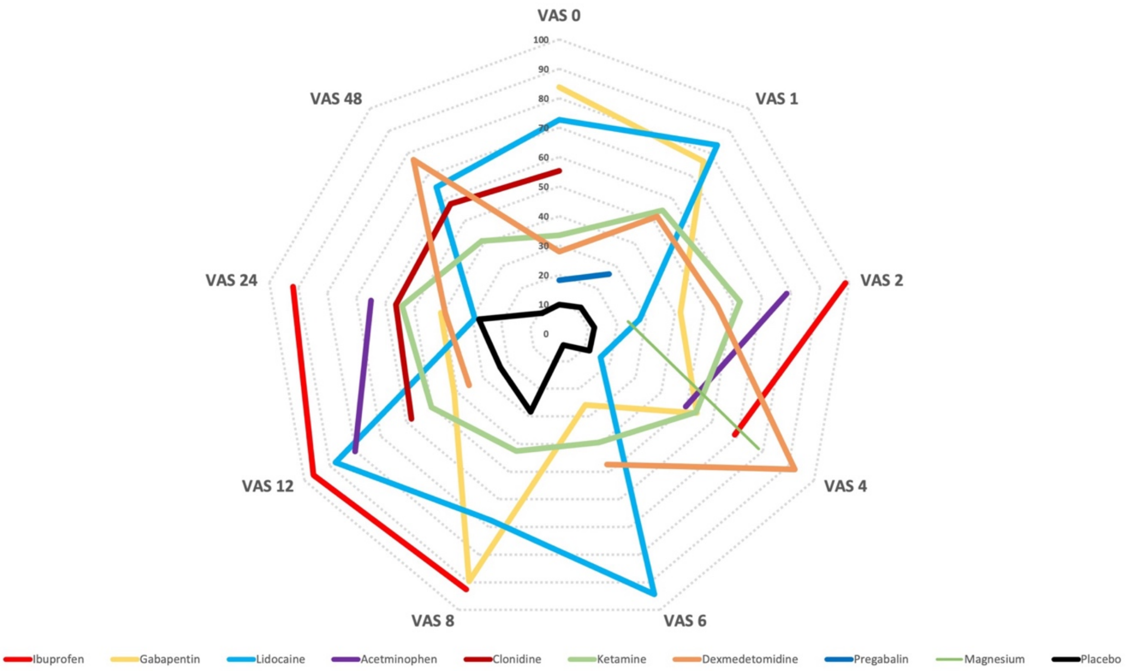

The image depicts a radar (or spider) chart illustrating the SUCRA (Surface Under the Cumulative Ranking) probabilities for various intravenous non-opioid analgesics and adjuvants used in multimodal anesthesia to reduce Visual Analogue Scale (VAS) score across multiple time points. In the legend of the figure, each line represents a different treatment (e.g., ibuprofen, gabapentin, lidocaine, etc.), and each axis stands for a different time point following treatment (VAS 0, VAS 1, etc.). The further from the center a line extends on an axis, the higher the SUCRA value for that treatment at that time point, indicating its relative effectiveness. Placebo is included as a reference for comparison. This visual representation allows for a comparative analysis of the efficacy of each treatment over time, providing a clear overview of which treatments perform better or worse at specific post-treatment intervals.

Fig.S2. SUCRA ranking for impact of intravenous non-opioid analgesics and adjuvants on rescue analgesic treatment at different time points

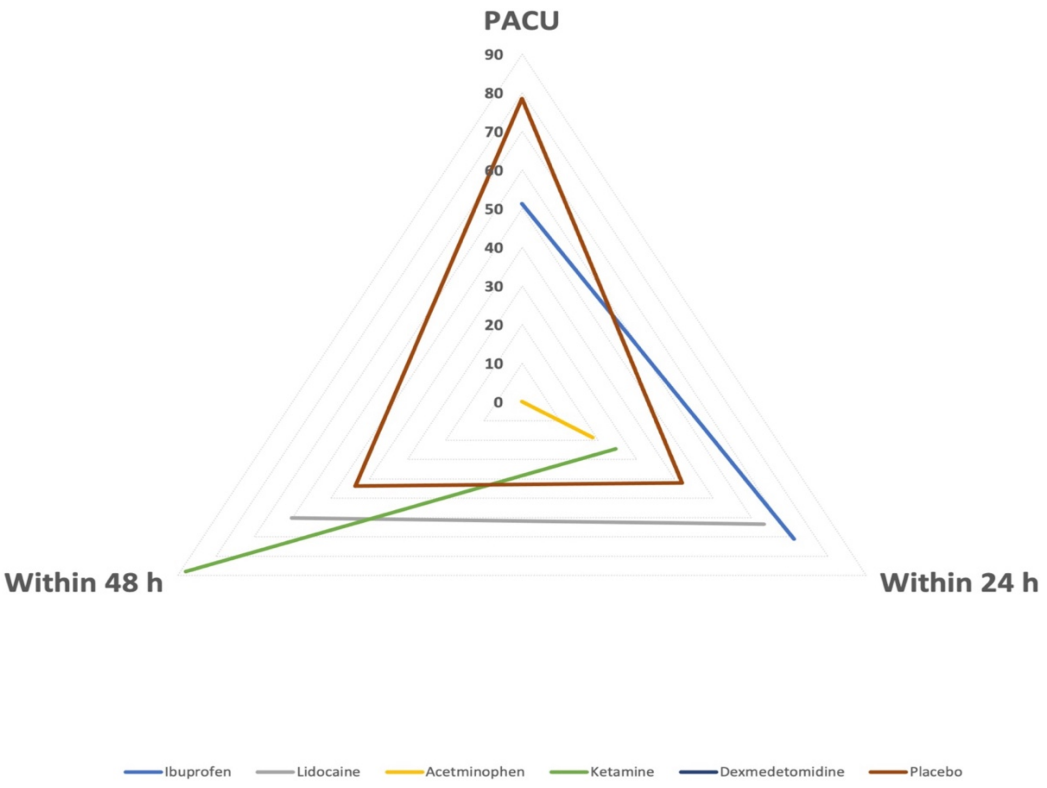

The image depicts a radar (or spider) chart illustrating the SUCRA (Surface Under the Cumulative Ranking) probabilities for various intravenous non-opioid analgesics and adjuvants used in multimodal anesthesia to reduce rescue analgesic treatment across multiple time points. In the legend of the figure, each line represents a different treatment (e.g., ibuprofen, lidocaine, etc.), and each axis stands for a different time point following treatment (PACU, within 24 h after surgery, within 48 h after surgery). The further from the center a line extends on an axis, the higher the SUCRA value for that treatment at that time point, indicating its relative effectiveness. Placebo is included as a reference for comparison. This visual representation allows for a comparative analysis of the efficacy of each treatment over time, providing a clear overview of which treatments perform better or worse at specific post-treatment intervals.

Fig.S3. SUCRA ranking for impact of intravenous non-opioid analgesics and adjuvants on Postoperative Nausea and Vomiting (PONV) at different time points

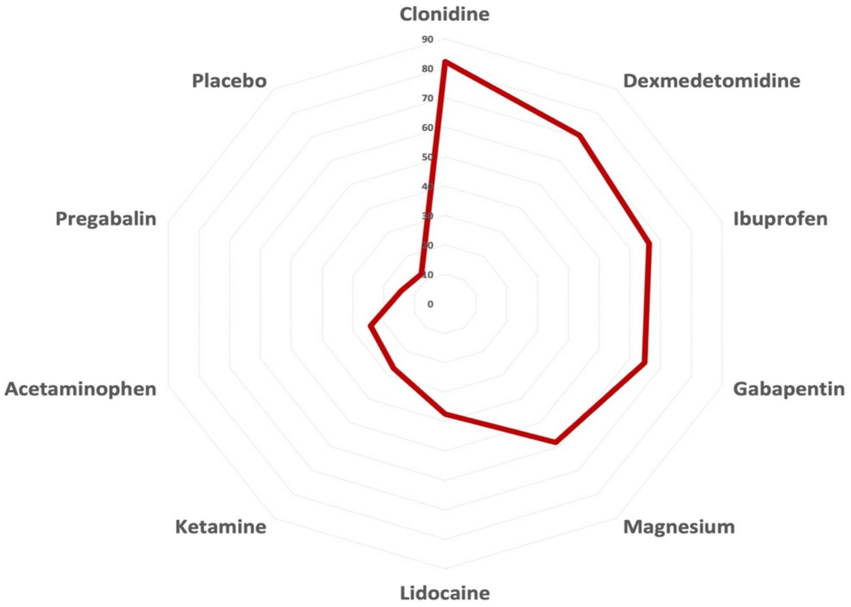

The image presents a radar chart depicting the SUCRA (Surface Under the Cumulative Ranking) probabilities for various intravenous non-opioid analgesics and adjuvants used in multimodal anesthesia for the prevention of nausea and vomiting (PONV) in postoperative period. Each axis corresponds to a specific treatment, with the proximity to the outer edge indicating greater efficacy in rescue treatment reduction. Treatments nearer to the perimeter demonstrate higher effectiveness, while those towards the center are less beneficial. The chart provides a visual comparison of the relative benefits of each treatment in rescue treatment prevention.
