# Supplementary material for: Non-Opioid Analgesics and Adjuvants after Surgery in Adults with Obesity: Systematic Review with Network Meta-Analysis of Randomized Controlled Trials
Source: J Clin Med. 2024 Apr 3;13(7):2100. doi: 10.3390/jcm13072100 (PMC11012569; doi:10.3390/jcm13072100)
Supplement: Supplementary file 1 [file jcm-13-02100-s001.zip › SMC_JCM_R1/SMC2. Study characteristics. 02.03.24.pdf]

Study characteristics

The characteristics of the Randomized Controlled Trials (RCTs) included in the network meta-analysis are reported in the following table.

Table 1. Characteristics of studies considered for review and meta-analysis

Study (year)	Country	Surgery	Anesthesia (Drug)	Population (BMI) (Treatment/ Control)	Treatment (Drug - Dose)	Control (Drug -Dose)	Primary Endpoint	Metanalysis endpoints (PAIN-PONV-QoR40)
**Bakhamees (2007)**	Egypt	Laparoscopic RYGB	Propofol (TIVA), Fentanyl, lidocaine	**Obese** (40/40)	Dexmedetomidine 0,8µg/kg (TBW) + 0,4 µg/kg/h	Placebo	Intraoperative hemodinamic control, anesthetic and analgesic requirements	VAS (60 min, 2h), PONV
**Kamal (2008)**	Egypt	Upper abdominal surgery	Midazolam, Isoflurane, fentanyl	**Obese** (40/40)	Ketamine Postoperative PCA pump, 0,015 mg/kg (IBW) boluses	Placebo	Postoperative opioid consumption	Rescue 48h, PONV
**Tufanogullari (2008)**	United States	Laparoscopic bariatric surgery	Desflurane, fentanyl, lidocaine IV, bupivacaine wound infiltration	**Morbidly obese** (20/20)	Dexmedetomidine 0.2 µg/kg/h	Placebo	Anesthetic and analgesic requirements	VAS (0 min, 30 min, 60 min, 90 min, 24h, 48h, POD 7), Rescue (24h, 48h), PONV
**Tufanogullari (2008)**	United States	Laparoscopic bariatric surgery	Desflurane, fentanyl, lidocaine IV, bupivacaine wound infiltration	**Morbidly obese** (20/20)	Dexmedetomidine 0.4 µg/kg/h	Placebo	Anesthetic and analgesic requirements	VAS (0 min, 30 min, 60 min, 90 min, 24h, 48h, POD 7), Rescue (24h, 48h), PONV
**Tufanogullari (2008)**	United States	Laparoscopic bariatric surgery	Desflurane, fentanyl, lidocaine IV, bupivacaine wound infiltration	**Morbidly obese** (20/20)	Dexmedetomidine 0.8 µg/kg/h	Placebo	Anesthetic and analgesic requirements	VAS (0 min, 30 min, 60 min, 90 min, 24h, 48h, POD 7), Rescue (24h, 48h), PONV
**Sollazzi (2009)**	Italy	Open biliopancreatic diversion	Sevoflurane, Fentanyl, ketorolac	**Morbidly obese** (23/27)	Preinduction Ketamine 0,5 mg/kg (IBW) + Clonidine 3 µg/kg (IBW)	Placebo	Posptoperative pain scores and opioid consuption	VAS (0 min, 30 min, 60 min, 6h, 12h)
**Schulmeyer (2010)**	Chile	LSG	Isoflurane, remifentanil, morphine, ketoprofen	**Obese** (39/41)	Pregabalin 150 mg preoperatory	Placebo	Postoperative pain scores and opioid consuption	PONV
**Hasanein (2011)**	Egypt	Laparoscopic RYGBP	Propofol (TIVA), Remifentanil, lidocaine	**Morbidly obese** (30/30)	Ketamine 1 µg/kg/min	Placebo	Hemodynamic stability, postoperative analgesic requirement, and recovery profile	VAS (60 min, 2h), PONV

**De Oliveira (2014)**	United States	Laparoscopic gastric reduction surgery	Midazolam, desflurane, hydromorphone, remifentanil, Bupivacaine wound infiltration	**Obese** (24/26)	Lidocaine 1,5 mg/kg (DBW) + 2 mg/kg/h	Placebo	Postoperative quality of recovery	PONV, QoR40 POD 1
**Naja (2014)**	Lebanon	LSG	Midazolam, Sevoflurane, Nitrous Oxide, Fentanyl	**Obese (BMI≥30)** (30/30)	Dexmedetomidine 0,5-0,8 µg/kg/h	Clonidine 0,8-1,2 µg/kg	Posptoperative pain scores and analgesic consuption	VAS (0 min, 6h, 12h, 24h) , Rescue (6h, 24h), PONV
**Hassani (2015)**	Iran	Laparoscopic Gastric Bypass	Midazolam, Propofol (TIVA), Fentanyl	**Obese (BMI≥40)** (30/30)	Gabapentin 100 mg preoperatory	Placebo	Postoperative pain scores	VAS (0 min, 60 min, 2h, 4h, 6h), PONV
**Salama (2016)**	Egypt	LSG	Sevoflurane, fentanyl, Bupivacaine wound infiltration	**Morbidly obese** (30/30)	Pregabalin 75 mg + dexmedetomidine 0,5 µg/kg + 0,4 µg/kg/h	Placebo	Posptoperative pain scores and analgesic consuption	PONV
**El Chaar (2016)**	United States	Laparoscopic RYGBP or LSG	Intravenous or inhalatory and morphine	**Obese (BMI≥35)** (50/50)	Acetaminophen 1g preoperative + 1g q6	Placebo	Total hospital network direct costs	VAS 24h
**Sherif (2017)**	Egypt	LSG	Sevoflurane, Fentanyl	**Obese (BMI≥35)** (46/49)	Lidocaine 1,5 mg/kg (LBW) + 2 mg/kg/h	Placebo	Postoperative morphine consumption	PONV, QoR40 POD 3
**Sherif (2017)**	Egypt	LSG	Sevoflurane, Fentanyl	**Obese (BMI≥35)** (49/49)	Dexmedetomidine 1 µg/kg (LBW) + 0,4 µg/kg/h	Placebo	Postoperative morphine consumption	PONV, QoR40 POD 3
**Sherif (2017)**	Egypt	LSG	Sevoflurane, Fentanyl	**Obese (BMI≥35)** (49/46)	Dexmedetomidine 1 µg/kg (LBW) + 0,4 µg/kg/h	Lidocaine 1,5 mg/kg (LBW) + 2 mg/kg/h	Postoperative morphine consumption	PONV, QoR40 POD 3
**Cooke (2018)**	United States	Minimally invasive sleeve gastrectomy	Desflurane, fentanyl or hydromorphone, lidocaine, ketorolac, bilateral TAP block (bupivacaine + dexamethasone)	**Obese (BMI≥30)** (64/63)	Acetaminophen 1g intraoperative + 1g q6	Placebo	Hospital length of stay	VAS (0 min, 24h, 48h), Rescue PACU, PONV
**Erdogan Kayhan (2018)**	Turkey	Laparoscopic RYGBP	Desflurane, morphine, remifentanil	**Obese** (30/30)	Ibuprofen 800 mg intraoperative + 1g q8	Acetaminophen 1g intraoperative + 1g q8	Postoperative opioid consumption	VAS 24h, Rescue (PACU, 24h), PONV
**Lange (2018)**	United States	Laparoscopic RYGBP	Intravenous or inhalatory and not specified opioid	**Obese (BMI≥35)** (44/45)	Acetaminophen Postoperative 1g q6	Placebo	Hospital LOS	Rescue 24h

**Martins (2018)**	Brazil	Bariatric non laparoscopic surgery	Sevoflurane, sufentanil, metamizole, ketoprofen, Bupivacaine wound infiltration	**Obese** (30/30)	Pregabalin 75 mg preoperatory	Placebo	Postoperative quality of recovery	VAS (0 min, 60 min, 24h), Rescue (PACU, 24h) PONV, QoR40 POD 1
**Mostafa (2018)**	Egypt	LSG	Sevoflurane, fentanyl	**Obese non diabetic (35≤BMI≤55)** (30/30)	Dexmedetomidine 1 µg/kg (LBW) + 0,5 µg/kg/h	Placebo	Variation of perioperative serial blood sugar levels	VAS (30 min, 60 min, 2h, 3h, 6h, 12h, 24h), PONV
**Rupniewska-Ladyko (2018)**	Poland	LSG	Desflurane, Fentanyl, metamizole	**Obese** (57/56)	Gabapentin 1200 mg preoperatory	Placebo	Time to first postoperative analgesic requirement	VAS (4h, 12h), PONV
**CIftci (2019)**	Turkey	LSG	Sevoflurane, Fentanyl, remifentanil, meperidine	**Obese (BMI≥35)** (30/30)	Acetaminophen 1g intraoperative + 1g q8	Placebo	Postoperative pain scores and opioid consuption	VAS (0 min, 2h, 4h, 8h, 12h, 24h), PONV
**CIftci (2019)**	Turkey	LSG	Sevoflurane, Fentanyl, remifentanil, meperidine	**Obese (BMI≥35)** (30/30)	Ibuprofen 800 mg intraoperative + 1g q8	Placebo	Postoperative pain scores and opioid consuption	VAS (0 min, 2h, 4h, 8h, 12h, 24h), PONV
**CIftci (2019)**	Turkey	LSG	Sevoflurane, Fentanyl, remifentanil, meperidine	**Obese (BMI≥35)** (30/30)	Ibuprofen 800 mg intraoperative + 1g q8	Acetaminophen 1g intraoperative + 1g q8	Postoperative pain scores and opioid consuption	VAS (0 min, 2h, 4h, 8h, 12h, 24h), PONV
**El Mourad (2019)**	Egypt	LSG	Isoflurane, Fentanyl, Bupivacaine wound infiltration	**Obese (BMI≥35)** (40/40)	Magnesium 30 mg/kg	Placebo	Intraoperative hemodynamic stability	PONV
**Khan (2019)**	Saudi Arabia	LSG	Desflurane, Fentanyl, remifentanil	**Obese (30≤BMI≤50)** (25/25)	Gabapentin 1200 mg preoperatory	Placebo	Postoperative pain scores and opioid consuption	VAS (0 min, 4h, 8h), Rescue 6h, PONV
**Ranganathan (2018)**	United States	Laparoscopic RYGB	Intravenous or inhalatory and not specified opioid	**Obese** (26/20)	Dexmedetomidine 1µg/kg	Placebo	Posptoperative pain scores and opioid consuption	VAS 4h
**Wang (2019)**	United States	Laparoscopic Gastric Bypass or LSG	Intravenous or inhalatory and not specified opioid	**Obese** (44/46)	Ketamine Postoperative, 0,4 mg/kg	Placebo	Postoperative pain scores	PONV
**De Oliveira (2020)**	Brazil	Laparoscopic Gastric Bypass	Midazolam, Sevoflurane, fentanyl, Ropivacaine wound infiltration	**Obese** (29/29)	Lidocaine 1,5 mg/kg pre induction + 2 mg/kg/h	Placebo	Postoperative pain scores	VAS (0 min, 60 min, 2h, 4h, 6h, 12h, 24h), PONV
**Jabbour (2020)**	Lebanon	Open OAGB	Sevoflurane, Remifentanil, Nitrous Oxide, Morphine, Acetaminophen	**Morbidly obese** (20/20)	Ketamine 0,2 mg/kg + 0,15 mg/kg/h	Placebo	Posptoperative pain scores and opioid consuption	Rescue 24h

**Jabbour (2020)**	Lebanon	Open OAGB	Sevoflurane, Remifentanil, Nitrous Oxide, Morphine, Acetaminophen	**Morbidly obese** (20/20)	Ketamine(0,2 mg/kg + 0,15 mg/kg/h) + Magnesium (50 mg/kg + 8 mg/kg/h)	Placebo	Posptoperative pain scores and opioid consuption	Rescue 24h
**Jabbour (2020)**	Lebanon	Open OAGB	Sevoflurane, Remifentanil, Nitrous Oxide, Morphine, Acetaminophen	**Morbidly obese** (20/20)	Ketamine(0,2 mg/kg + 0,15 mg/kg/h) + Magnesium (50 mg/kg + 8 mg/kg/h)	Ketamine 0,2 mg/kg + 0,15 mg/kg/h	Posptoperative pain scores and opioid consuption	Rescue 24h
**Kasputyte (2020)**	Lithuania	Bariatric Surgery	Sevoflurane, Fentanyl, remifentanil, morphine, Acetaminophen, ketoprofen, Bupivacaine wound infiltration	**Obese** (15/17)	Ketamine 0,15 mg/kg (LBW)	Placebo	Postoperative opioid consumption	VAS (30 min, 60 min, 90 min, 2h), PONV
**Sakata (2020)**	Brazil	Laparoscopic Gastric Bypass	Midazolam, Sevoflurane, fentanyl, Ropivacaine wound infiltration	**Obese** (29/29)	Lidocaine 1,5 mg/kg pre induction + 2 mg/kg/h	Placebo	Time of recovery of gastrointestinal function	PONV
**Adhikary (2021)**	United States	LSG	Midazolam, desflurane, fentanyl	**Obese** (34/37)	Ketamine 0,5 mg/kg + Magnesium 2g	Placebo	Postoperative opioid consumption	VAS (0 min, 4h, 8h, 12h, 24h)
**Adhikary (2021)**	United States	LSG	Midazolam, desflurane, fentanyl	**Obese** (34/37)	Ketamine 0,5 mg/kg + Magnesium 2g	Ketamine 0,5 mg/kg	Postoperative opioid consumption	VAS (0 min, 4h, 8h, 12h, 24h)
**Adhikary (2021)**	United States	LSG	Midazolam, desflurane, fentanyl	**Obese** (37/37)	Ketamine 0,5 mg/kg	Placebo	Postoperative opioid consumption	VAS (0 min, 4h, 8h, 12h, 24h)
**Mehta (2021)**	United States	Laparoscopic RYGB	Fentanyl, hydromorphone, Acetaminophen, Lidocaine and Bupivacaine wound infiltration	**Morbidly obese** (27/27)	Ketamine 20 mg + 5μg/kg/min	Placebo	Postoperative opioid consumption	VAS 24h
**Plass (2021)**	France	Laparoscopic bariatric surgery	Desflurane, remifentanil, Ketamine, Nefopam, acetaminophen,parecox ib, ropivacaine wound infiltration	**Obese** (88/88)	Lidocaine 1,5 mg/kg (ABW) + 2 mg/kg/h (ABW)	Placebo	Postoperative opioid consumption	VAS (24h, 48h, 72h), Rescue (24h, 48h), PONV
**Seman (2021)**	United States	Laparoscopic Gastric Bypass	Midazolam, Sevoflurane, Fentanyl, Acetaminophen, Ketorolac	**Obese (BMI≥35)** (18/17)	Ketamine 0,3 mg/kg + 0,2 mg/kg/h (IBW) for 24h	Placebo	Postoperative opioid consumption	VAS (12h, 24h, 48h), PONV

**Sun (2022)**	China	Laparoscopic bariatric surgery	Midazolam, Propofol, sevoflurane, sufentanil, remifentanil, ketorolac	**Obese (BMI≥35)** (33/33)	Lidocaine 1,5 mg/kg (DBW) + 2 mg/kg/h	Placebo	Postoperative quality of recovery	PONV, QoR40 POD 1
**Uston (2022)**	Turkey	LSG	Desflurane, Remifentanil, Tramadol, Paracetamol, Ketoprofen	**Obese (BMI≥35)** (24/24)	Lidocaine 2 mg/kg/h (LBW) intraop + 1 mg/kg/h for 12h	Ketamine 0,5 mg/kg/h (IBW) intraop + 0,3 mg/kg/h for 12h	Postoperative pain scores in the first 12 hours	VAS (0 min, 60 min, 3h, 6h, 12h, 24h, 48h), PONV
**Uston (2022)**	Turkey	LSG	Desflurane, Remifentanil, Tramadol, Paracetamol, Ketoprofen	**Obese (BMI≥35)** (25/24)	Dexmedetomidine 0,5 µg/kg/h (ABW) intraop + 0,3 µg/kg/h for 12h	Ketamine 0,5 mg/kg/h (IBW) intraop + 0,3 mg/kg/h for 12h	Postoperative pain scores in the first 12 hours	VAS (0 min, 60 min, 3h, 6h, 12h, 24h, 48h), PONV
**Uston (2022)**	Turkey	LSG	Desflurane, Remifentanil, Tramadol, Paracetamol, Ketoprofen	**Obese (BMI≥35)** (25/24)	Dexmedetomidine 0,5 µg/kg/h (ABW) intraop + 0,3 µg/kg/h for 12h	Lidocaine 2 mg/kg/h (LBW) intraop + 1 mg/kg/h for 12h	Postoperative pain scores in the first 12 hours	VAS (0 min, 60 min, 3h, 6h, 12h, 24h, 48h), PONV
**Ahmed (2023)**	Egypt	LSG	Desflurane, Fentanyl	**Obese (BMI≥35)** (30/30)	Magnesium 30 mg/kg + 20 mg/kg in the first 8h post	Placebo	Tissue perfusion	VAS (30 min, 90 min, 2h, 4h, 8h), PONV
**Khalil (2023)**	Egypt	LSG	Midazolam, Sevoflurane, Fentanyl, lidocaine IV	**Obese (BMI≥35)** (30/30)	Dexmedetomidine 0,5 µg/kg (IBW) + 0,5 µg/kg/h	Placebo	Postoperative morphine consumption	VAS (0 min, 30 min, 60 min, 2h, 6h, 12h, 24h), PONV
**Khalil (2023)**	Egypt	LSG	Midazolam, Sevoflurane, Fentanyl, lidocaine IV	**Obese (BMI≥35)** (30/30)	Dexmedetomidine 0,5 µg/kg (IBW) + 0,5 µg/kg/h	Ketamine 0,3 mg/kg (IBW) + 0,3 mg/kg/h	Postoperative morphine consumption	VAS (0 min, 30 min, 60 min, 2h, 6h, 12h, 24h), PONV
**Yang (2023)**	China	LSG	Midazolam, Propofol (TCI), Sufentanil, Remifentanil, Flurbiprofen, Dexmedetomidine	**Obese** (34/34)	Esketamine 0,2 mg/kg + 0,2 mg/kg/h	Placebo	Postoperative pain scores	VAS (30 min, 60 min, 2h, 6h, 12h, 24h, 48h)
**Yurttas (2023)**	Switzerland	Laparoscopic bariatric surgery	Propofol (TCI), fentanyl, remifentanil (TCI), Acetaminophen	**Obese** (68/69)	Lidocaine 1.5mg/kg (LBW) + 1,5 mg/kg/h for 4h	Placebo	Postoperative pain scores	VAS (60 min, 2h, 3h, 4h), PONV
**Zhang (2023)**	China	Laparoscopic bariatric surgery	Midazolam, Sevoflurane, Propofol, Sufentanil, Ketorolac	**Obese (BMI≥30)** (35/35)	Esketamine 0,5 mg/kg/h	Placebo	QoR40 score on POD 1	VAS (24h, 48h, POD 7), PONV, QoR40 POD 1

BMI: Body Mass Index; DBW (dosing body weight); IBW. Ideal Body Weight; LBW. Lean Body Weight; ABW. Adjusted Body Weight; LSG: Laparoscopic Sleeve Gastrectomy; OAGB: One Anastomosis Gastric Bypass; PACU: Post-Anesthesia Care Unit; PCA: Patient Controlled Analgesia; PONV: Postoperative Nausea and Vomiting; POD: Postoperative Day; 3QoR-40: Quality of Recovery-40, a questionnaire; RYGB: Roux-en-Y Gastric Bypass; TCI: Target Controlled Infusion; VAS: Visual Analog Scale.
