# Supplementary material for: Non-Opioid Analgesics and Adjuvants after Surgery in Adults with Obesity: Systematic Review with Network Meta-Analysis of Randomized Controlled Trials
Source: J Clin Med. 2024 Apr 3;13(7):2100. doi: 10.3390/jcm13072100 (PMC11012569; doi:10.3390/jcm13072100)
Supplement: Supplementary file 1 [file jcm-13-02100-s001.zip › SMC_JCM_R1/SMC4. RoB2 assessment. Reasons for the RoB2 judgements. 02.03.24 .pdf]

Risk of bias assessment based on revised Cochrane risk-of-bias tool for randomized trials (RoB 2).

Below, the reasons for the RoB 2 assessment of each Randomized Controlled Trial (RCT) included in the network meta-analysis are presented. The RoB 2 tool evaluates five key domains of bias risk: the randomization process, deviations from intended interventions, missing outcome data, measurement of the outcome, selection of the reported result, and an overall RoB 2 assessment. Within each domain, a series of questions (referred to as 'signaling questions') are posed to identify potential sources of bias. Based on the answers to these signaling questions, an assessment of bias for each domain is proposed, ranging from 'low' or 'high' risk to 'some concerns'. The overall RoB 2 assessment is then derived from these domain-specific evaluations.

Adhikary SD (2021)

Entry	Judgment	Description
**Randomization process**	*Low risk*	Block randomization using a computer-generated random allocation sequence reported. Allocation sequence concealed described.
**Deviations from intended interventions**	*Low risk*	Anesthesiologist delivering the interventions not aware of participants' assigned intervention. The patients and outcome assessor not aware of the participants' assigned interventions.
**Missing outcome data**	*Low risk*	Data about outcomes available for all participants. Result not biased by missing outcome data.
**Measurement of outcome**	*Low risk*	Measuring outcomes appropriated. Outcome assessor not aware of the intervention received by study participants. Group allocation revealed after data collection and analysis.
**Selection of reported result**	*Low risk*	Trial analyzed in accordance with a prespecified plan.
**Overall RoB2**	*Low risk*	

Ahmed YIA (2023)

Entry	Judgment	Description
**Randomization process**	*Some concerns*	Randomization method not reported. Allocation sequence concealed described. No apparent imbalances.
**Deviations from intended interventions**	*Low risk*	Anesthesiologist delivering the interventions not aware of participants' assigned intervention. The patients and outcome assessor not aware of the participants' assigned interventions.
**Missing outcome data**	*Low risk*	Data about outcomes available for all participants. Result not biased by missing outcome data.
**Measurement of outcome**	*Low risk*	Measuring outcomes appropriated. Outcome assessor not aware of the intervention received by study participants.
**Selection of reported result**	*Low risk*	Trial analyzed in accordance with a prespecified plan.
**Overall RoB2**	*Some concerns*	

Bakhamees HS (2007)

Entry	Judgment	Description
**Randomization process**	*Some concerns*	Randomization method and concealment were not described. No apparent imbalances.
**Deviations from intended interventions**	*Some concerns*	Anesthesiologist delivering the interventions aware of participants' assigned intervention. The patients and the PACU nurses not aware of the participants' assigned interventions. No apparent imbalances.
**Missing outcome data**	*Low risk*	Data about outcomes available for all participants. Result not biased by missing outcome data.
**Measurement of outcome**	*Low risk*	Measuring outcomes appropriated. Outcome assessor not aware of the intervention received by study participants.
**Selection of reported result**	*Low risk*	Trial analyzed in accordance with a prespecified plan.
**Overall RoB2**	*Some concerns*	

Ciftci B (2019)

Entry	Judgment	Description
**Randomization process**	Some concerns	Randomization method and concealment not described. No apparent imbalances.
**Deviations from intended interventions**	Low risk	Anesthesiologist delivering the interventions not aware of participants' assigned intervention. The patients and outcome assessor not aware of the participants' assigned interventions.
**Missing outcome data**	Low risk	Data about outcomes available for all participants. Result not biased by missing outcome data.
**Measurement of outcome**	Low risk	Measuring outcomes appropriated. Outcome assessor not aware of the intervention received by study participants.
**Selection of reported result**	Low risk	Trial analyzed in accordance with a prespecified plan.
**Overall RoB2**	Some concerns	

Cooke FE (2018)

Entry	Judgment	Description
**Randomization process**	*Low risk*	Block randomization using a computer-generated random allocation sequence reported. Allocation sequence concealed described.
**Deviations from intended interventions**	*Low risk*	Anesthesiologist delivering the interventions, outcome assessor, patient and data analyst not aware of participants' assigned intervention.
**Missing outcome data**	*Low risk*	Data about outcomes available for all participants. Result not biased by missing outcome data.
**Measurement of outcome**	*Low risk*	Measuring outcomes appropriated. Outcome assessor not aware of participants' assigned intervention.
**Selection of reported result**	*Low risk*	Trial analyzed in accordance with a prespecified plan.
**Overall RoB2**	*Low risk*	

De Oliveira Jr. GS (2014)

Entry	Judgment	Description
**Randomization process**	Low risk	Randomization using a computer-generated random allocation sequence reported. Allocation sequence concealed described.
**Deviations from intended interventions**	Low risk	Anesthesiologist delivering the interventions, outcome assessor and patient not aware of participants' assigned intervention.
**Missing outcome data**	Low risk	Data about outcomes available for all participants. Result not biased by missing outcome data.
**Measurement of outcome**	Low risk	Measuring outcomes appropriated. Outcome assessor not aware of participants' assigned intervention.
**Selection of reported result**	Low risk	Trial analyzed in accordance with a prespecified plan.
**Overall RoB2**	Low risk	

De Oliveira CMB (2020)

Entry	Judgment	Description
**Randomization process**	Low risk	Randomization using a computer-generated random allocation sequence reported. Allocation sequence concealed described.
**Deviations from intended interventions**	Low risk	Anesthesiologist delivering the interventions, outcome assessor, patient and data analyst not aware of participants' assigned intervention.
**Missing outcome data**	Low risk	Data about outcomes available for all participants. Result not biased by missing outcome data.
**Measurement of outcome**	Low risk	Measuring outcomes appropriated. Outcome assessor not aware of participants' assigned intervention.
**Selection of reported result**	Low risk	Trial analyzed in accordance with a prespecified plan.
**Overall RoB2**	Low risk	

El Chaar M (2016)

Entry	Judgment	Description
**Randomization process**	Low risk	Block randomization using a computer-generated random allocation sequence reported. Allocation sequence concealed described.
**Deviations from intended interventions**	Low risk	Anesthesiologist delivering the interventions, outcome assessor and patient not aware of participants' assigned intervention.
**Missing outcome data**	Low risk	Data about outcomes available for all participants. Result not biased by missing outcome data.
**Measurement of outcome**	Low risk	Measuring outcomes appropriated. Outcome assessor not aware of participants' assigned intervention.
**Selection of reported result**	Low risk	Trial analyzed in accordance with a prespecified plan.
**Overall RoB2**	Low risk	

El Mourad MB (2019)

Entry	Judgment	Description
**Randomization process**	Low risk	Randomization using a computer-generated random allocation sequence reported. Allocation sequence concealed described.
**Deviations from intended interventions**	Low risk	Anesthesiologist delivering the interventions, outcome assessor and patient not aware of participants' assigned intervention.
**Missing outcome data**	Low risk	Data about outcomes available for all participants. Result not biased by missing outcome data.
**Measurement of outcome**	Low risk	Measuring outcomes appropriated. Outcome assessor not aware of participants' assigned intervention.
**Selection of reported result**	Low risk	Trial analyzed in accordance with a prespecified plan.
**Overall RoB2**	Low risk	

Erdogan Kayhan G (2018)

Entry	Judgment	Description
**Randomization process**	Low risk	Block randomization using a computer-generated random allocation sequence reported. Allocation sequence concealed described.
**Deviations from intended interventions**	Low risk	Anesthesiologist delivering the interventions, outcome assessor and patient not aware of participants' assigned intervention.
**Missing outcome data**	Low risk	Data about outcomes available for all participants. Result not biased by missing outcome data.
**Measurement of outcome**	Low risk	Measuring outcomes appropriated. Outcome assessor not aware of participants' assigned intervention.
**Selection of reported result**	Low risk	Trial analyzed in accordance with a prespecified plan.
**Overall RoB2**	Low risk	

Hasanein R (2011)

Entry	Judgment	Description
**Randomization process**	Some concerns	Randomization method and concealment not described. No apparent imbalances.
**Deviations from intended interventions**	Some concerns	Anesthesiologist delivering the interventions aware of participants' assigned intervention. The patients and outcome assessor not aware of the participants' assigned interventions. No apparent imbalances.
**Missing outcome data**	Low risk	Data about outcomes available for all participants. Result not biased by missing outcome data.
**Measurement of outcome**	Low risk	Measuring outcomes appropriated. Outcome assessor not aware of the intervention received by study participants.
**Selection of reported result**	Low risk	Trial analyzed in accordance with a prespecified plan.
**Overall RoB2**	Some concerns	

Hassani V (2015)

Entry	Judgment	Description
**Randomization process**	Some concerns	Block randomization reported. Allocation sequence concealed not described. No apparent imbalances.
**Deviations from intended interventions**	Low risk	Anesthesiologist delivering the interventions not aware of participants' assigned intervention. The patients and outcome assessor not aware of the participants' assigned interventions.
**Missing outcome data**	Low risk	Data about outcomes available for all participants. Result not biased by missing outcome data.
**Measurement of outcome**	Low risk	Measuring outcomes appropriated. Outcome assessor not aware of the intervention received by study participants.
**Selection of reported result**	Low risk	Trial analyzed in accordance with a prespecified plan.
**Overall RoB2**	Some concerns	

Jabbour H (2020)

Entry	Judgment	Description
**Randomization process**	Low risk	Randomization using a computer-generated random allocation sequence reported. Allocation sequence concealed described. No apparent imbalances.
**Deviations from intended interventions**	Low risk	Anesthesiologist delivering the interventions not aware of participants' assigned intervention. The patients and outcome assessor not aware of the participants' assigned interventions.
**Missing outcome data**	Low risk	Data about outcomes available for all participants. Result not biased by missing outcome data.
**Measurement of outcome**	Low risk	Measuring outcomes appropriated. Outcome assessor not aware of the intervention received by study participants.
**Selection of reported result**	Low risk	Trial analyzed in accordance with a prespecified plan.
**Overall RoB2**	Low risk	

Kamal HM (2008)

Entry	Judgment	Description
**Randomization process**	Some concerns	Randomization method not described. Allocation sequence concealed reported. No apparent imbalances.
**Deviations from intended interventions**	Low risk	Anesthesiologist delivering anesthesia not involved in the study. The patients and outcome assessor not aware of the participants' assigned interventions. No apparent imbalances.
**Missing outcome data**	Low risk	Data about outcomes available for all participants. Result not biased by missing outcome data.
**Measurement of outcome**	Low risk	Measuring outcomes appropriated. Outcome assessor not aware of the intervention received by study participants.
**Selection of reported result**	Low risk	Trial analyzed in accordance with a prespecified plan.
**Overall RoB2**	Some concerns	

Kasputytė G (2020)

Entry	Judgment	Description
**Randomization process**	Some concerns	Randomization method and concealment not described. No apparent imbalances.
**Deviations from intended interventions**	Some concerns	Not specified whether anesthesiologist delivering the interventions and/or patients and/or outcome assessor are not aware of the participants' assigned interventions. No apparent imbalances.
**Missing outcome data**	Low risk	Data about outcomes available for all participants. Result not biased by missing outcome data.
**Measurement of outcome**	Low risk	Measuring outcomes appropriated.
**Selection of reported result**	Low risk	Trial analyzed in accordance with a prespecified plan.
**Overall RoB2**	Some concerns	

Khalil BNM (2023)

Entry	Judgment	Description
**Randomization process**	Low risk	Randomization using a computer-generated random allocation sequence reported. Allocation sequence concealed described. No apparent imbalances.
**Deviations from intended interventions**	Low risk	Anesthesiologist delivering anesthesia not involved in the study. The patients and outcome assessor not aware of the participants' assigned interventions.
**Missing outcome data**	Low risk	Data about outcomes available for all participants. Result not biased by missing outcome data.
**Measurement of outcome**	Low risk	Measuring outcomes appropriated. Outcome assessor not aware of the intervention received by study participants.
**Selection of reported result**	Low risk	Trial analyzed in accordance with a prespecified plan.
**Overall RoB2**	Low risk	

Khan MU (2019)

Entry	Judgment	Description
**Randomization process**	Some concerns	Randomization using a computer-generated random allocation sequence reported. Allocation sequence concealed described. No apparent imbalances.
**Deviations from intended interventions**	Low risk	Anesthesiologist delivering anesthesia not involved in the study. The patients and outcome assessor not aware of the participants' assigned interventions.
**Missing outcome data**	Low risk	Data about outcomes available for all participants. Result not biased by missing outcome data.
**Measurement of outcome**	Low risk	Measuring outcomes appropriated. Outcome assessor not aware of the intervention received by study participants.
**Selection of reported result**	Low risk	Trial analyzed in accordance with a prespecified plan.
**Overall RoB2**	Low risk	

Lange M (2018)

Entry	Judgment	Description
**Randomization process**	Low risk	Randomization reported. Allocation sequence concealed described.
**Deviations from intended interventions**	Low risk	Anesthesiologist delivering anesthesia not aware of participants' assigned intervention. The patients and outcome assessor not aware of the participants' assigned interventions.
**Missing outcome data**	Low risk	Data about outcomes available for all participants. Result not biased by missing outcome data.
**Measurement of outcome**	Low risk	Measuring outcomes appropriated. Outcome assessor not aware of the intervention received by study participants.
**Selection of reported result**	Low risk	Trial analyzed in accordance with a prespecified plan.
**Overall RoB2**	Low risk	

Martins MJ (2018)

Entry	Judgment	Description
**Randomization process**	Low risk	Randomization using a computer-generated random allocation sequence. Allocation sequence concealed reported.
**Deviations from intended interventions**	Low risk	Anesthesiologist delivering anesthesia not involved in the study. The patients and outcome assessor not aware of the participants' assigned interventions.
**Missing outcome data**	Low risk	Data about outcomes available for all participants. Result not biased by missing outcome data.
**Measurement of outcome**	Low risk	Measuring outcomes appropriated. Outcome assessor not aware of the intervention received by study participants.
**Selection of reported result**	Low risk	Trial analyzed in accordance with a prespecified plan.
**Overall RoB2**	Low risk	

Mehta SD (2020)

Entry	Judgment	Description
**Randomization process**	Some concerns	Randomization method and concealment not described. No apparent imbalances.
**Deviations from intended interventions**	Low risk	Anesthesiologist delivering anesthesia not involved in the study. The patients and outcome assessor not aware of the participants' assigned interventions.
**Missing outcome data**	Low risk	Data about outcomes available for all participants. Result not biased by missing outcome data.
**Measurement of outcome**	Low risk	Measuring outcomes appropriated. Outcome assessor not aware of the intervention received by study participants.
**Selection of reported result**	Low risk	Trial analyzed in accordance with a prespecified plan.
**Overall RoB2**	Some concerns	

Mostafa RH (2018)

Entry	Judgment	Description
**Randomization process**	Some concerns	Randomization using a computer-generated random allocation sequence reported. Allocation sequence concealed not described. No apparent imbalances.
**Deviations from intended interventions**	Some concerns	Not specified whether anesthesiologist delivering the interventions and/or patients and/or outcome assessor are not aware of the participants' assigned interventions. No apparent imbalances.
**Missing outcome data**	Low risk	Data about outcomes available for all participants. Result not biased by missing outcome data.
**Measurement of outcome**	Low risk	Measuring outcomes appropriated. Outcome assessor not aware of the intervention received by study participants.
**Selection of reported result**	Low risk	Trial analyzed in accordance with a prespecified plan.
**Overall RoB2**	Some concerns	

Naja ZM (2014)

Entry	Judgment	Description
**Randomization process**	Some concerns	Randomization method was not reported. Allocation sequence concealed described. No apparent imbalances.
**Deviations from intended interventions**	Low risk	Anesthesiologist delivering anesthesia not aware of participants' assigned intervention. The patients and outcome assessor not aware of the participants' assigned interventions.
**Missing outcome data**	Low risk	Data about outcomes available for all participants. Result not biased by missing outcome data.
**Measurement of outcome**	Low risk	Measuring outcomes appropriated. Outcome assessor not aware of the intervention received by study participants.
**Selection of reported result**	Low risk	Trial analyzed in accordance with a prespecified plan.
**Overall RoB2**	Some concerns	

Plass F (2020)

Entry	Judgment	Description
**Randomization process**	*Low risk*	Block randomization using a computer-generated random allocation sequence reported. Allocation sequence concealed described.
**Deviations from intended interventions**	*Low risk*	Anesthesiologist delivering the interventions not aware of participants' assigned intervention. The patients and outcome assessor not aware of the participants' assigned interventions.
**Missing outcome data**	*Low risk*	Data about outcomes available for all participants. Result not biased by missing outcome data.
**Measurement of outcome**	Low risk	Measuring outcomes appropriated. Outcome assessor not aware of the intervention received by study participants.
**Selection of reported result**	Low risk	Trial analyzed in accordance with a prespecified plan.
**Overall RoB2**	Low risk	

Ranganathan P (2019)

Entry	Judgment	Description
**Randomization process**	*Low risk*	Block randomization using a computer-generated random allocation sequence reported. Allocation sequence concealed r described.
**Deviations from intended interventions**	*Low risk*	Anesthesiologist delivering the interventions not aware of participants' assigned intervention. The patients and outcome assessor not aware of the participants' assigned interventions.
**Missing outcome data**	*Low risk*	Data about outcomes available for all participants. Result not biased by missing outcome data.
**Measurement of outcome**	Low risk	Measuring outcomes appropriated. Outcome assessor not aware of the intervention received by study participants.
**Selection of reported result**	Low risk	Trial analyzed in accordance with a prespecified plan.
**Overall RoB2**	Low risk	

Rupniewska-Ladyko A (2018)

Entry	Judgment	Description
**Randomization process**	*Low risk*	Block randomization using a computer-generated random allocation sequence reported. Allocation sequence concealed described.
**Deviations from intended interventions**	*Low risk*	Anesthesiologist delivering the interventions not aware of participants' assigned intervention. The patients and outcome assessor not aware of the participants' assigned interventions.
**Missing outcome data**	*Low risk*	Data about outcomes available for all participants. Result not biased by missing outcome data.
**Measurement of outcome**	Low risk	Measuring outcomes appropriated. Outcome assessor not aware of the intervention received by study participants.
**Selection of reported result**	Low risk	Trial analyzed in accordance with a prespecified plan.
**Overall RoB2**	Low risk	

Sakata RK (2020)

Entry	Judgment	Description
**Randomization process**	*Low risk*	Randomization using a computer-generated random allocation sequence reported. Allocation sequence concealed described.
**Deviations from intended interventions**	*Low risk*	Anesthesiologist delivering the interventions not aware of participants' assigned intervention. The patients and outcome assessor not aware of the participants' assigned interventions.
**Missing outcome data**	*Low risk*	Data about outcomes available for all participants. Result not biased by missing outcome data.
**Measurement of outcome**	Low risk	Measuring outcomes appropriated. Outcome assessor not aware of the intervention received by study participants.
**Selection of reported result**	Low risk	Trial analyzed in accordance with a prespecified plan.
**Overall RoB2**	Low risk	

Salama AK (2019)

Entry	Judgment	Description
**Randomization process**	*Low risk*	Randomization using a computer-generated random allocation sequence reported. Allocation sequence concealed described.
**Deviations from intended interventions**	*Low risk*	Anesthesiologist delivering the interventions not aware of participants' assigned intervention. The patients and outcome assessor not aware of the participants' assigned interventions.
**Missing outcome data**	*Low risk*	Data about outcomes available for all participants. Result not biased by missing outcome data.
**Measurement of outcome**	Low risk	Measuring outcomes appropriated. Outcome assessor not aware of the intervention received by study participants.
**Selection of reported result**	Low risk	Trial analyzed in accordance with a prespecified plan.
**Overall RoB2**	Low risk	

Schulmeyer Cabrera MC (2010)

Entry	Judgment	Description
**Randomization process**	Some concerns	Randomization using a computer-generated random allocation sequence reported. Allocation sequence concealed not described. No apparent imbalances.
**Deviations from intended interventions**	Low risk	Anesthesiologist delivering anesthesia not aware of participants' assigned intervention. The patients and outcome assessor not aware of the participants' assigned interventions.
**Missing outcome data**	Low risk	Data about outcomes available for all participants. Result not biased by missing outcome data.
**Measurement of outcome**	Low risk	Measuring outcomes appropriated. Outcome assessor not aware of the intervention received by study participants.
**Selection of reported result**	Low risk	Trial analyzed in accordance with a prespecified plan.
**Overall RoB2**	Some concerns	

Seman TM (2021)

Entry	Judgment	Description
**Randomization process**	Some concerns	Randomization method and concealment not described. No apparent imbalances.
**Deviations from intended interventions**	Some concerns	Not specified whether anesthesiologist delivering the interventions and/or patients and/or outcome assessor not aware of the participants' assigned interventions. No apparent imbalances.
**Missing outcome data**	Low risk	Data about outcomes available for all participants. Result not biased by missing outcome data.
**Measurement of outcome**	Some concerns	Measuring outcomes appropriated. Not specified whether outcome assessor not aware of the participants' assigned interventions. No apparent imbalances.
**Selection of reported result**	Low risk	Trial analyzed in accordance with a prespecified plan.
**Overall RoB2**	Some concerns	

Sherif AA (2017)

Entry	Judgment	Description
**Randomization process**	*Low risk*	Randomization using a computer-generated random allocation sequence reported. Allocation sequence concealed described.
**Deviations from intended interventions**	*Low risk*	Anesthesiologist delivering the interventions not aware of participants' assigned intervention. The patients and outcome assessor not aware of the participants' assigned interventions.
**Missing outcome data**	*Low risk*	Data about outcomes available for all participants. Result not biased by missing outcome data.
**Measurement of outcome**	Low risk	Measuring outcomes appropriated. Outcome assessor not aware of the intervention received by study participants.
**Selection of reported result**	Low risk	Trial analyzed in accordance with a prespecified plan.
**Overall RoB2**	Low risk	

Sollazzi L (2009)

Entry	Judgment	Description
**Randomization process**	Some concerns	Randomization method was not reported. Allocation sequence concealed described. No apparent imbalances.
**Deviations from intended interventions**	Some concerns	Anesthesiologist delivering the interventions aware of participants' assigned intervention. The patients and outcome assessor aware of the participants' assigned interventions. No apparent imbalances.
**Missing outcome data**	Low risk	Data about outcomes available for all participants. Result not biased by missing outcome data.
**Measurement of outcome**	Some concerns	Measuring outcomes appropriated. Outcome assessor aware of the intervention received by study participants. No apparent imbalances.
**Selection of reported result**	Low risk	Trial analyzed in accordance with a prespecified plan.
**Overall RoB2**	Some concerns	

Sun J (2022)

Entry	Judgment	Description
**Randomization process**	*Low risk*	Randomization using a computer-generated random allocation sequence reported. Allocation sequence concealed described.
**Deviations from intended interventions**	Some concerns	Anesthesiologist delivering the interventions aware of participants' assigned intervention. The patients and outcome assessor aware of the participants' assigned interventions. No apparent imbalances.
**Missing outcome data**	*Low risk*	Data about outcomes available for all participants. Result not biased by missing outcome data.
**Measurement of outcome**	*Low risk*	Measuring outcomes appropriated. Outcome assessor not aware of the intervention received by study participants.
**Selection of reported result**	*Low risk*	Trial analyzed in accordance with a prespecified plan.
**Overall RoB2**	*Some concerns*	

Tufanogullari B (2008)

Entry	Judgment	Description
**Randomization process**	*Low risk*	Randomization using a computer-generated random allocation sequence reported. Allocation sequence concealed described.
**Deviations from intended interventions**	*Low risk*	Anesthesiologist delivering the interventions not aware of participants' assigned intervention. The patients and outcome assessor not aware of the participants' assigned interventions.
**Missing outcome data**	*Low risk*	Data about outcomes available for all participants. Result not biased by missing outcome data.
**Measurement of outcome**	Low risk	Measuring outcomes appropriated. Outcome assessor not aware of the intervention received by study participants.
**Selection of reported result**	Low risk	Trial analyzed in accordance with a prespecified plan.
**Overall RoB2**	Low risk	

Ustun YB (2022)

Entry	Judgment	Description
**Randomization process**	*Low risk*	Randomization using a computer-generated random allocation sequence reported. Allocation sequence concealed described.
**Deviations from intended interventions**	*Low risk*	Anesthesiologist delivering the interventions not aware of participants' assigned intervention. The patients and outcome assessor not aware of the participants' assigned interventions.
**Missing outcome data**	*Low risk*	Data about outcomes available for all participants. Result not biased by missing outcome data.
**Measurement of outcome**	Low risk	Measuring outcomes appropriated. Outcome assessor not aware of the intervention received by study participants.
**Selection of reported result**	Low risk	Trial analyzed in accordance with a prespecified plan.
**Overall RoB2**	Low risk	

Wang J (2018)

Entry	Judgment	Description
**Randomization process**	*Low risk*	Randomization using a computer-generated random allocation sequence reported. Allocation sequence concealed described.
**Deviations from intended interventions**	*Low risk*	Anesthesiologist delivering the interventions not aware of participants' assigned intervention. The patients and outcome assessor not aware of the participants' assigned interventions.
**Missing outcome data**	*Low risk*	Data about outcomes available for all participants. Result not biased by missing outcome data.
**Measurement of outcome**	Low risk	Measuring outcomes appropriated. Outcome assessor not aware of the intervention received by study participants.
**Selection of reported result**	Low risk	Trial analyzed in accordance with a prespecified plan.
**Overall RoB2**	Low risk	

Yang T (2023)

Entry	Judgment	Description
**Randomization process**	*Low risk*	Randomization using a computer-generated random allocation sequence reported. Allocation sequence concealed described.
**Deviations from intended interventions**	*Low risk*	Anesthesiologist delivering the interventions not aware of participants' assigned intervention. The patients and outcome assessor not aware of the participants' assigned interventions.
**Missing outcome data**	*Low risk*	Data about outcomes available for all participants. Result not biased by missing outcome data.
**Measurement of outcome**	Low risk	Measuring outcomes appropriated. Outcome assessor not aware of the intervention received by study participants.
**Selection of reported result**	Low risk	Trial analyzed in accordance with a prespecified plan.
**Overall RoB2**	Low risk	

Yurttas T (2023)

Entry	Judgment	Description
**Randomization process**	*Low risk*	Block randomization using a computer-generated random allocation sequence reported. Allocation sequence concealed described.
**Deviations from intended interventions**	*Low risk*	Anesthesiologist delivering the interventions not aware of participants' assigned intervention. The patients and outcome assessor not aware of the participants' assigned interventions.
**Missing outcome data**	*Low risk*	Data about outcomes available for all participants. Result not biased by missing outcome data.
**Measurement of outcome**	Low risk	Measuring outcomes appropriated. Outcome assessor not aware of the intervention received by study participants.
**Selection of reported result**	Low risk	Trial analyzed in accordance with a prespecified plan.
**Overall RoB2**	Low risk	

Zhang J (2023)

Entry	Judgment	Description
**Randomization process**	*Low risk*	Randomization using a computer-generated random allocation sequence reported. Allocation sequence concealed described.
**Deviations from intended interventions**	*Low risk*	Anesthesiologist delivering the interventions not aware of participants' assigned intervention. The patients and outcome assessor not aware of the participants' assigned interventions.
**Missing outcome data**	*Low risk*	Data about outcomes available for all participants. Result not biased by missing outcome data.
**Measurement of outcome**	Low risk	Measuring outcomes appropriated. Outcome assessor not aware of the intervention received by study participants.
**Selection of reported result**	Low risk	Trial analyzed in accordance with a prespecified plan.
**Overall RoB2**	Low risk	
