# Supplementary material for: Non-Opioid Analgesics and Adjuvants after Surgery in Adults with Obesity: Systematic Review with Network Meta-Analysis of Randomized Controlled Trials
Source: J Clin Med. 2024 Apr 3;13(7):2100. doi: 10.3390/jcm13072100 (PMC11012569; doi:10.3390/jcm13072100)
Supplement: Supplementary file 1 [file jcm-13-02100-s001.zip › SMC_JCM_R1/SMC3. RoB2 assessment. "Traffic lightΓÇ¥ plot. 02.03.24.pdf]

SUPPLEMENTARY MATERIAL CONTENT (SMC) 3   RISK OF BIAS ASSESSMENT (ROB 2)

“Traffic light” plots of the domain-level judgements for each individual result

The following figure presents a "Risk of Bias" (RoB) 2 traffic light plot to visually represent the risk of bias assessment of included randomized controlled trials (RCTs). Each row represents a different study, with columns representing specific bias domains, such as bias from the randomization process (D1), deviations from intended interventions (D2), missing outcome data (D3), measurement of the outcome (D4), and selection of the reported result (D5). The 'Overall' column gives a summary judgement for each study. The color-coded symbols indicate the level of risk of bias for each domain: green for low risk, yellow for unclear risk, and red for high risk.

SUPPLEMENTARY MATERIAL CONTENT (SMC) 3 RISK OF BIAS ASSESSMENT (ROB 2)

Risk of bias domains

Study	D1	D2	D3	D4	D5	Overall
Adhikary SD (2021)	+	+	+	+	+	+
Ahmed YIA (2023)	-	+	+	+	+	-
Bakhamees HS (2007)	-	-	+	+	+	-
Ciftci B (2019)	-	+	+	+	+	-
Cooke FE (2018)	+	+	+	+	+	+
De Oliveira Jr. GS (2014)	+	+	+	+	+	+
De Oliveira CMB (2020)	+	+	+	+	+	+
El Mourad MB (2019)	+	+	+	+	+	+
Erdogan Kayhan G (2018)	+	+	+	+	+	+
Hasanein R (2011)	-	-	+	+	+	-
Hassani V (2015)	-	+	+	+	+	-
Jabbour H (2020)	+	+	+	+	+	+
Kamal HM (2008)	-	+	+	+	+	-
Kasputytė G (2020)	-	-	+	+	+	-
Khalil BNM (2023)	+	+	+	+	+	+
Khan MU (2019)	+	+	+	+	+	+
Lange M (2018)	+	+	+	+	+	+
Martins MJ (2018)	+	+	+	+	+	+
Mehta SD (2020)	-	+	+	+	+	-
Mostafa RH (2018)	-	-	+	+	+	-
Naja ZM (2014)	-	+	+	+	+	-
Plass F (2020)	+	+	+	+	+	+
Ranganathan P (2019)	+	+	+	+	+	+
Rupniewska-Ladyko A (2018)	+	+	+	+	+	+
Sakata RK (2020)	+	+	+	+	+	+
Salama AK (2019)	+	+	+	+	+	+
Schulmeyer Cabrera MC (2010)	-	+	+	+	+	-
Seman TM (2021)	-	-	+	+	+	-
Sherif AA (2017)	+	+	+	+	+	+
Sollazzi L (2009)	-	-	+	-	+	-
Sun J (2022)	+	-	+	+	+	-
Tufanogullari B (2008)	+	+	+	+	+	+
Ustun YB (2022)	+	+	+	+	+	+
Wang J (2018)	+	+	+	+	+	+
Yang T (2023)	+	+	+	+	+	+
Yurttas T (2023)	+	+	+	+	+	+
Zhang J (2023)	+	+	+	+	+	+

Domains:
D1: Bias arising from the randomization process.
D2: Bias due to deviations from intended interventio
D3: Bias due to missing outcome data.
D4: Bias in measurement of the outcome.
D5: Bias in selection of the reported result.

Judgement
- Some concerns
+ Low
