# Supplementary material for: Non-Opioid Analgesics and Adjuvants after Surgery in Adults with Obesity: Systematic Review with Network Meta-Analysis of Randomized Controlled Trials
Source: J Clin Med. 2024 Apr 3;13(7):2100. doi: 10.3390/jcm13072100 (PMC11012569; doi:10.3390/jcm13072100)
Supplement: Supplementary file 1 [file jcm-13-02100-s001.zip › SMC_JCM_R1/SMC5. Network graphs. 26.03.24.pdf]

Network Graphs Generated From Network Meta-Analysis for Different Time Points and Various Variables Considered

Below, a series of network graphs generated from a network meta-analysis, each corresponding to different time points and various variables considered (Postoperative Nausea and Vomiting [PONV], Use of Rescue Analgesics, Quality of Recovery-40 [QoR-40]), are presented. Within these graphs, the nodes represent the various interventions or studies analyzed, while the lines (or edges) illustrate the direct comparisons made between these interventions, factoring in the specified time points and variables. The thickness of the lines may denote the amount of evidence or the number of studies supporting each comparison, and the size of the nodes could reflect the total number of participants or the overall weight of each intervention in the analysis. This visual representation aids in understanding the interconnectedness of the data and the relative positions of interventions over time and across different outcome variables, providing a clear depiction of the landscape of evidence at various intervals.

Network Graphs Generated from Network Meta-Analysis for Various Time Points Considered

VAS (Visual Analog Scale) at the end of surgery

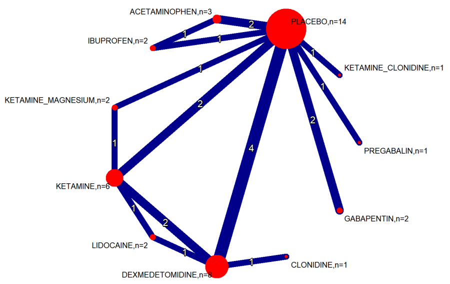

VAS at 30 minutes (after surgery)

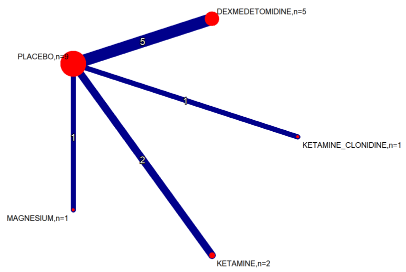

VAS at 30 minutes (after surgery)

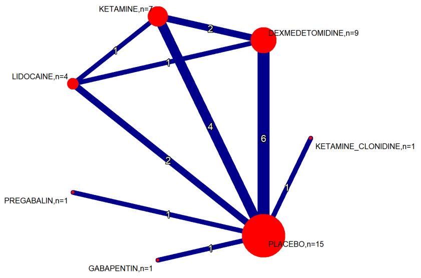

VAS at 2 hours (after surgery)

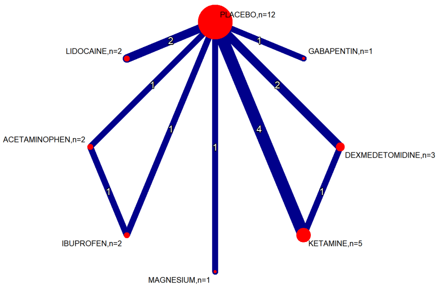

VAS at 4 hours (after surgery)

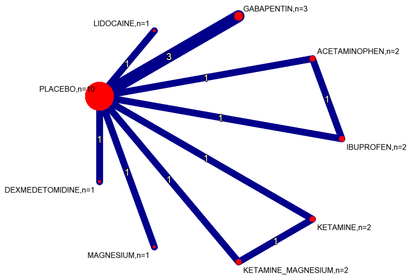

VAS at 6 hours (after surgery)

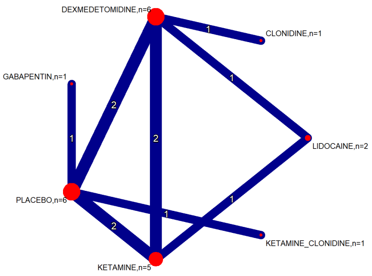

VAS at 8 hours (after surgery)

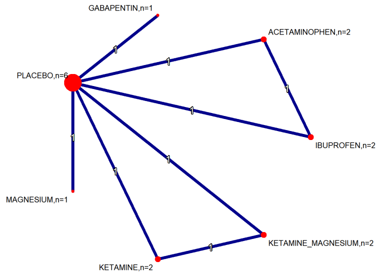

VAS at 12 hours (after surgery)

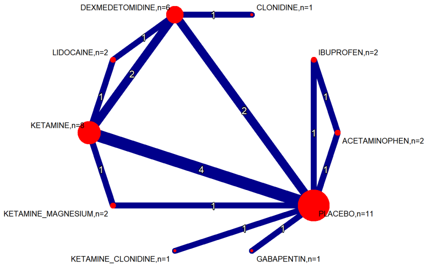

VAS at 24 hours (after surgery)

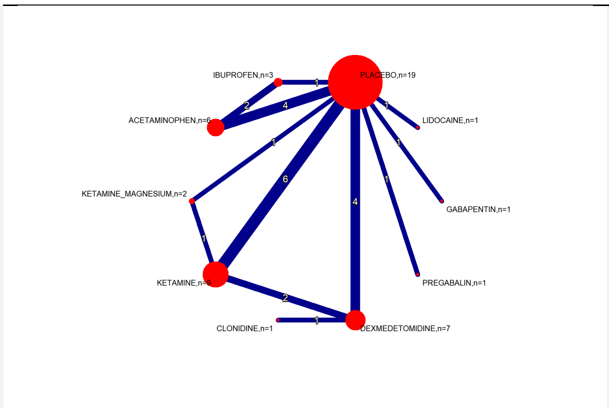

VAS at 48 hours (after surgery)

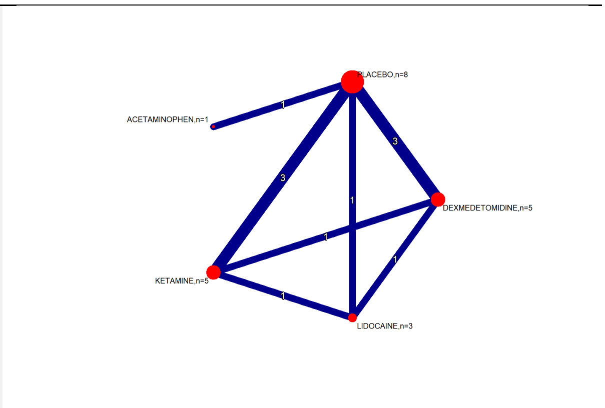

VAS at 7 days post-surgery

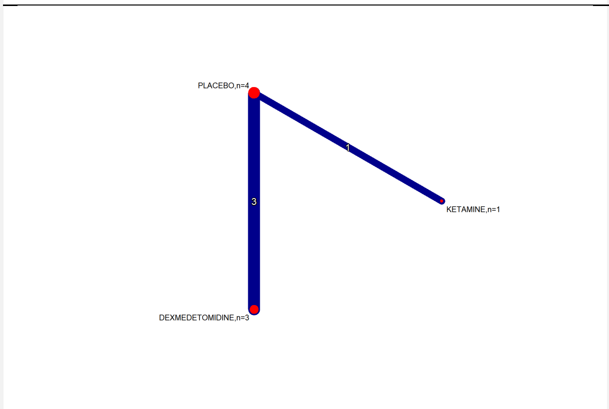

PONV

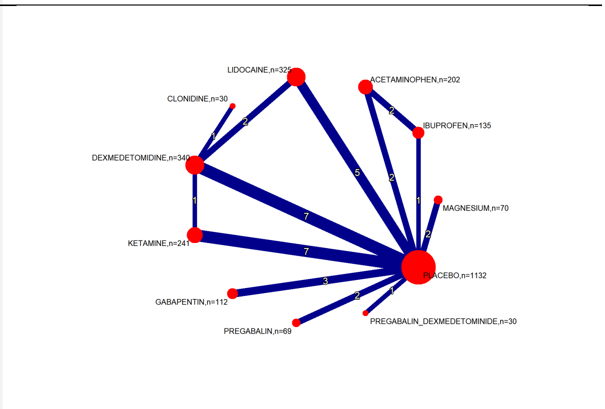

Rescue therapy during PACU stay

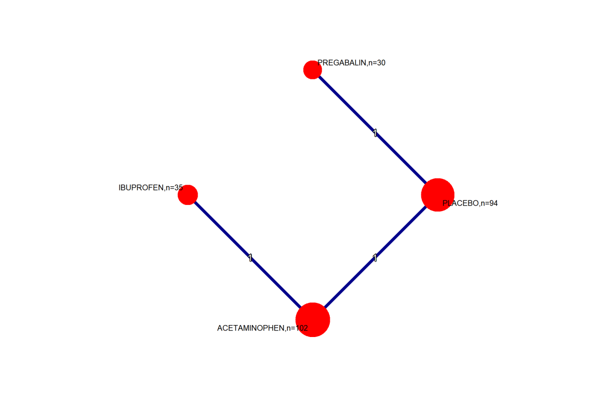

Rescue therapy within 6 hours (after surgery)

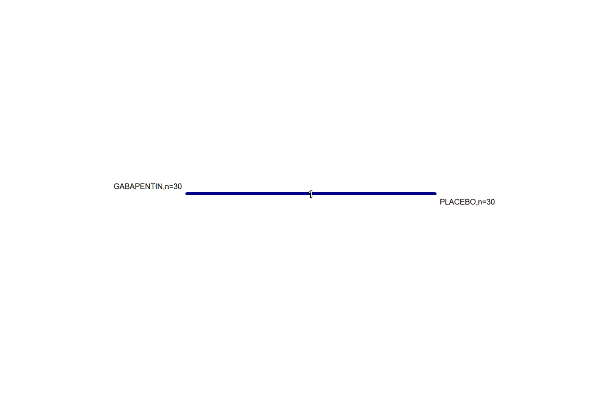

Rescue therapy within 24 hours (after surgery)

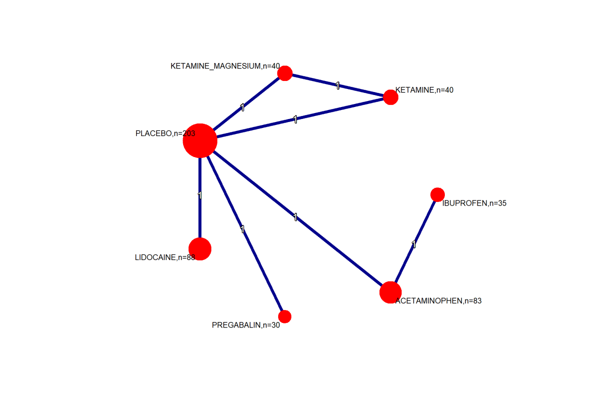

Rescue therapy within 48 hours (after surgery)

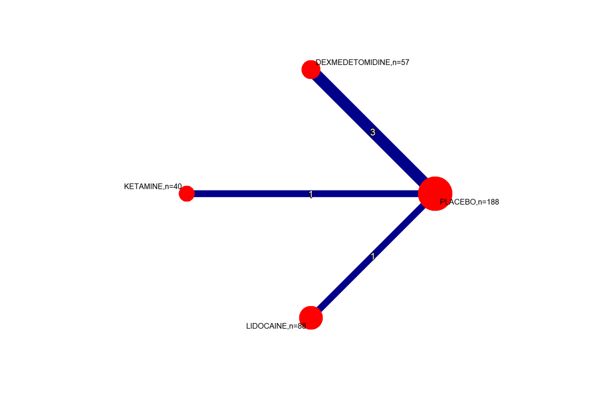

SUPPLEMENTARY MATERIAL CONTENT (SMC) 5 — Network Graphs

QoR40 on Postoperative Day 1		QoR40 on Postoperative Day 3	
	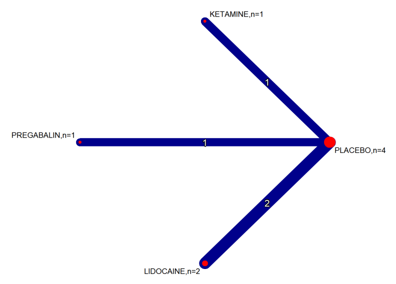		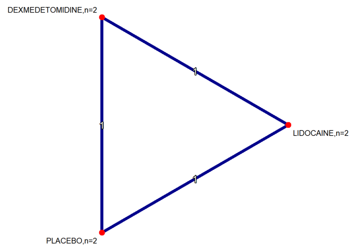
