# Supplementary material for: Non-Opioid Analgesics and Adjuvants after Surgery in Adults with Obesity: Systematic Review with Network Meta-Analysis of Randomized Controlled Trials
Source: J Clin Med. 2024 Apr 3;13(7):2100. doi: 10.3390/jcm13072100 (PMC11012569; doi:10.3390/jcm13072100)
Supplement: Supplementary file 1 [file jcm-13-02100-s001.zip › SMC_JCM_R1/SMC10. Separate indirect from direct evidence (SIDE). 04.03.24.pdf]

SUPPLEMENTARY MATERIAL CONTENT (SMC) 10 SIDE Analysis Heat Maps

Separate indirect from direct evidence (SIDE) Analysis Heat Maps in Network Meta-Analysis for Different Time Points and Various Variables Considered

Below, heat maps from a SIDE (Separate Indirect from Direct Evidence) analysis, crucial in network meta-analysis to discern consistency between direct and indirect evidence, are presented. Treatment comparisons are plotted on both axes, with color-coded cells indicating the degree of evidence discrepancy. Lighter shades suggest minimal discrepancy, while darker tones denote greater inconsistency. This visual tool is key for evaluating evidence reliability, highlighting areas of potential inconsistency, and aiding in the interpretation of meta-analytic results.

The generation of SIDE (Separate Indirect from Direct Evidence) heat maps relies on the presence of a closed evidence network, where treatments are compared both directly and indirectly. SIDE analysis requires a robust network with enough connections between nodes to properly assess the coherence between direct and indirect evidence. The absence of some SIDE heat maps is due to an incomplete evidence network, lacking adequate direct or indirect comparisons among all treatment options. Where connections are missing, SIDE heat maps cannot be produced, indicating data gaps necessary for evaluating the consistency of treatment effect estimates.

SUPPLEMENTARY MATERIAL CONTENT (SMC) 10 SIDE Analysis Heat Maps

VAS (Visual Analog Scale) at the end of surgery

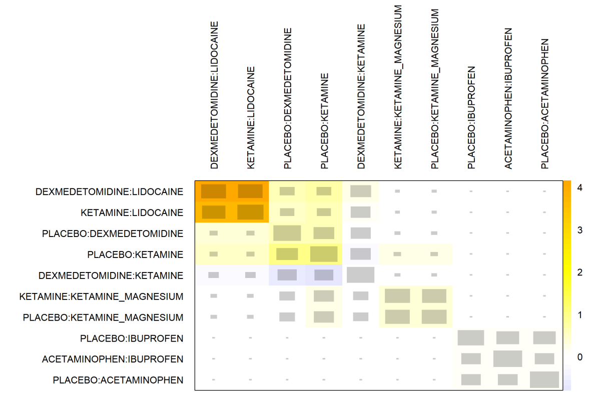

VAS at 30 minutes (after surgery)

Not available

VAS at 60 minutes (after surgery)

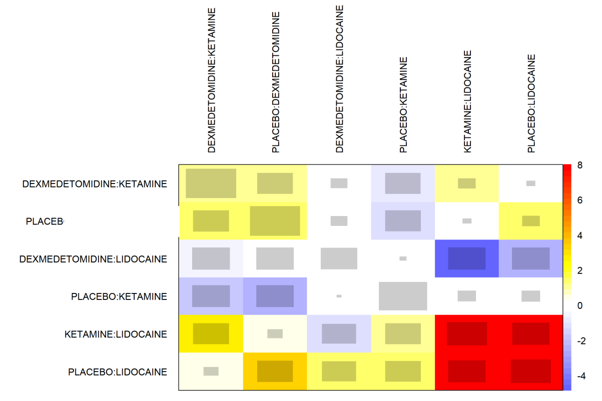

VAS at 2 hours (after surgery)

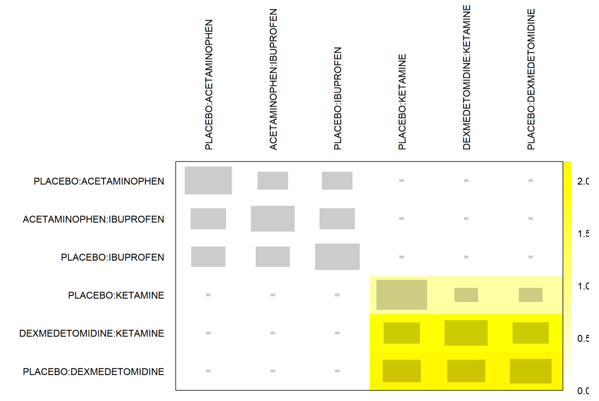

VAS at 4 hours (after surgery)

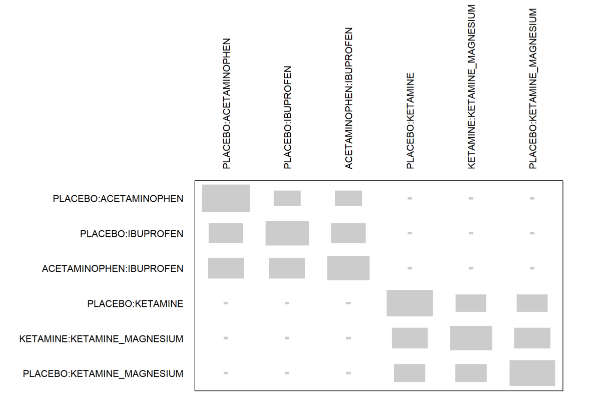

VAS at 6 hours (after surgery)

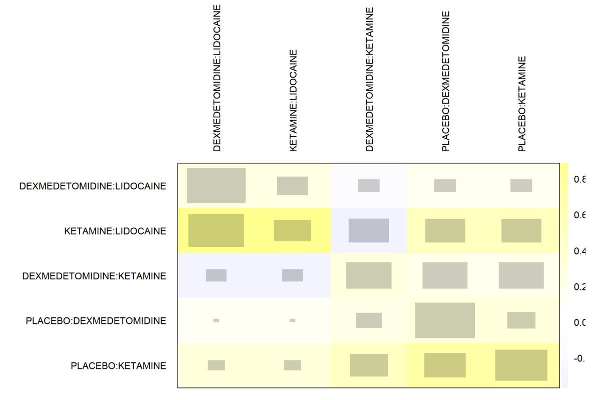

VAS at 8 hours (after surgery)

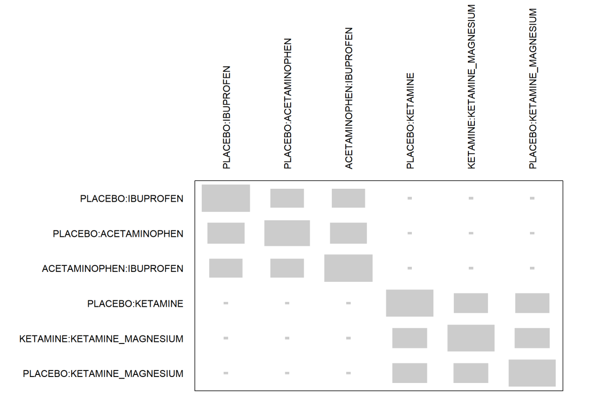

VAS at 12 hours (after surgery)

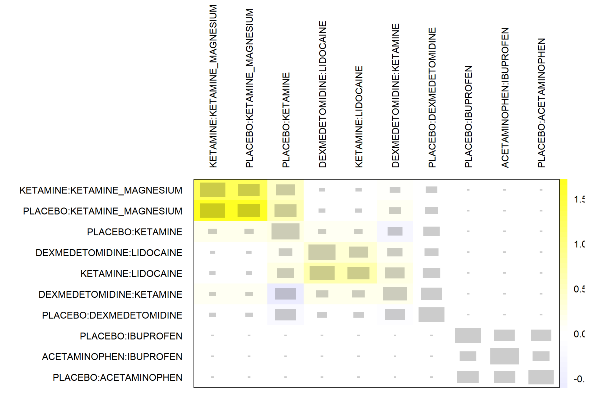

SUPPLEMENTARY MATERIAL CONTENT (SMC) 10 SIDE Analysis Heat Maps

VAS at 24 hours (after surgery)

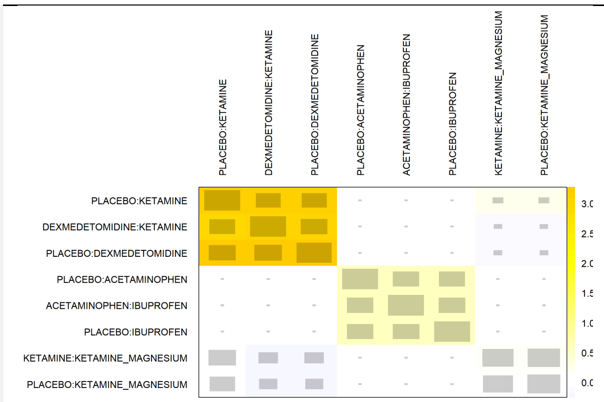

VAS at 48 hours (after surgery)

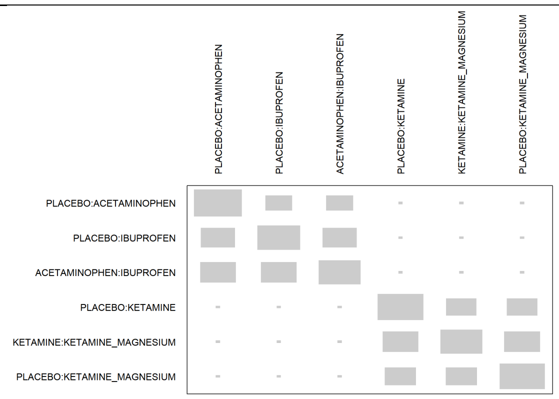

VAS at 7 days post-surgery

Not available

PONV

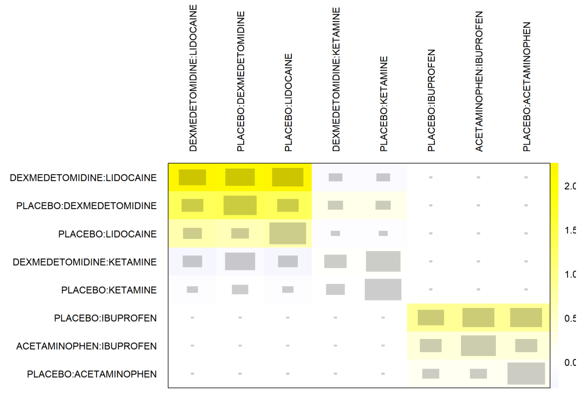

Rescue therapy during PACU stay

Not available

Rescue therapy within 6 hours (after surgery)

Not available

Rescue therapy within 24 hours (after surgery)

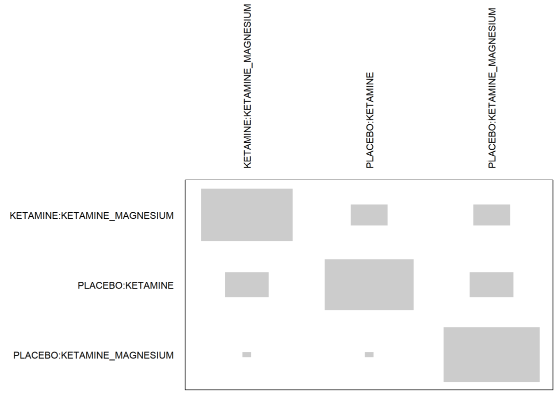

Rescue therapy within 48 hours (after surgery)

Not available

SUPPLEMENTARY MATERIAL CONTENT (SMC) 10 SIDE Analysis Heat Maps

QoR40 on Postoperative Day 1		QoR40 on Postoperative Day 3	
	**Not available**		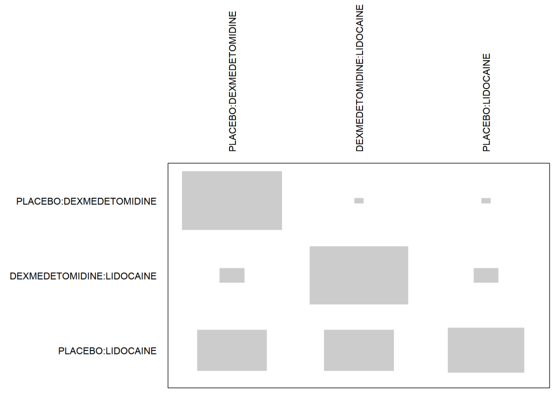
