# Supplementary material for: Non-Opioid Analgesics and Adjuvants after Surgery in Adults with Obesity: Systematic Review with Network Meta-Analysis of Randomized Controlled Trials
Source: J Clin Med. 2024 Apr 3;13(7):2100. doi: 10.3390/jcm13072100 (PMC11012569; doi:10.3390/jcm13072100)

Funnel Plots Derived from Network Meta-Analysis for Different Time Points and Various Variables Considered

Below, a series of funnel plots derived from a network meta-analysis are presented to show the potential risk of publication bias and the heterogeneity across the included studies, each corresponding to different time points of pain evaluation by the Visual Analog Scale (VAS), and other variables considered such as Postoperative Nausea and Vomiting (PONV), Use of Rescue Analgesics, and Quality of Recovery-40 (QoR-40). In these funnel plots, the Log Risk Ratios from individual studies are plotted against their standard errors. The standard errors are displayed on the vertical axis with a reversed scale, which places the larger, more robust studies at the top. The outer dashed lines define the triangular area where 95% of studies are expected to fall if there is no bias and no heterogeneity.

Funnel plots were utilized for visual inspection to assess the risk of publication bias in meta-analyses, whereas Egger's test for asymmetry was applied exclusively to analyses comprising 10 or more studies. A $p<0.1$ suggests possible risk of publication bias, whereas a $p\geq0.1$ indicates no substantial risk of publication bias.

.

SUPPLEMENTARY MATERIAL CONTENT (SMC) 7

Funnel plots

VAS (Visual Analog Scale) at the end of surgery

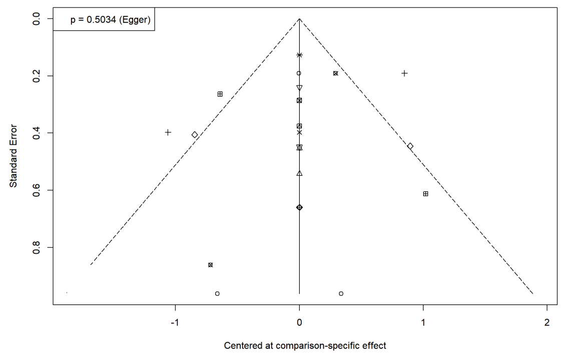

VAS at 30 minutes (after surgery)

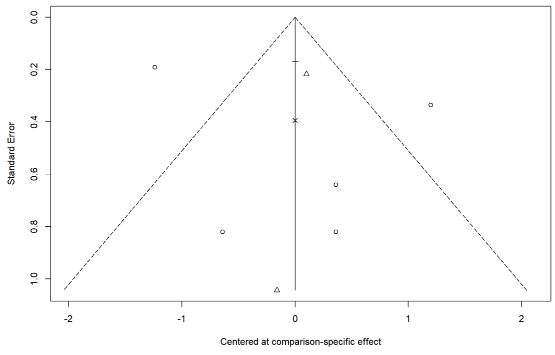

VAS at 60 minutes (after surgery)

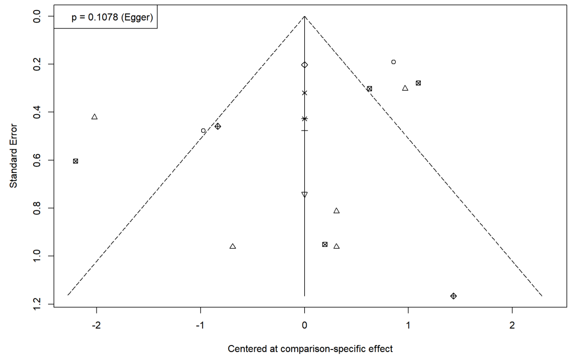

VAS at 2 hours (after surgery)

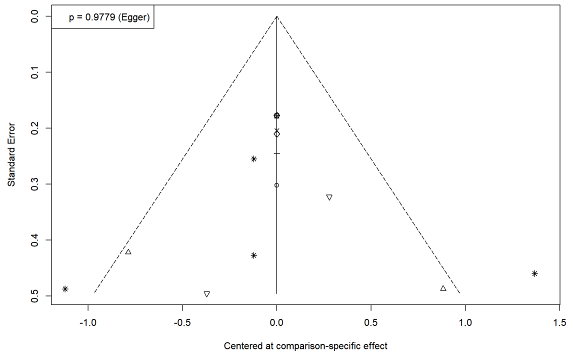

VAS at 4 hours (after surgery)

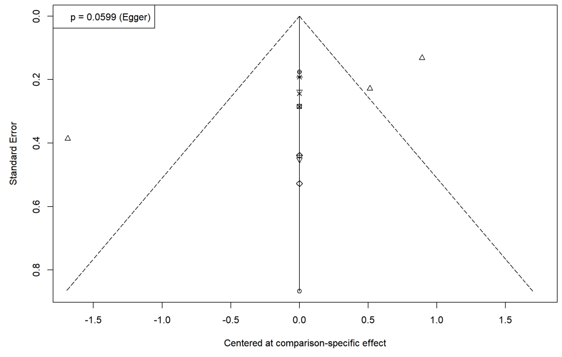

VAS at 6 hours (after surgery)

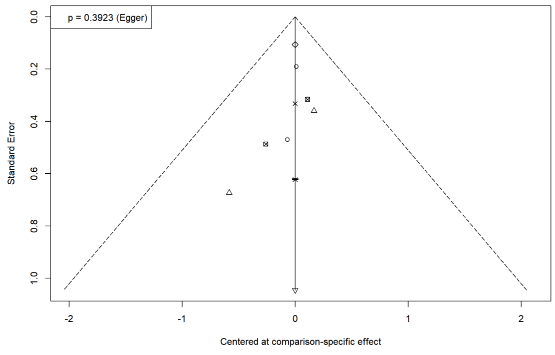

VAS at 8 hours (after surgery)

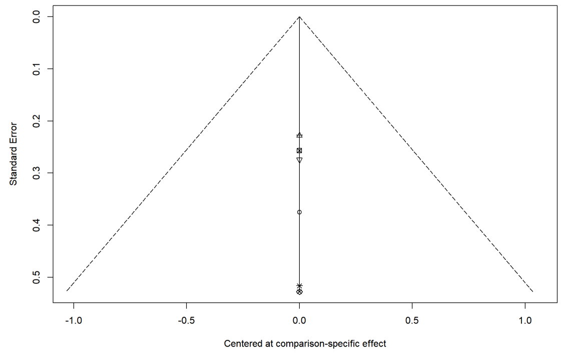

VAS at 12 hours (after surgery)

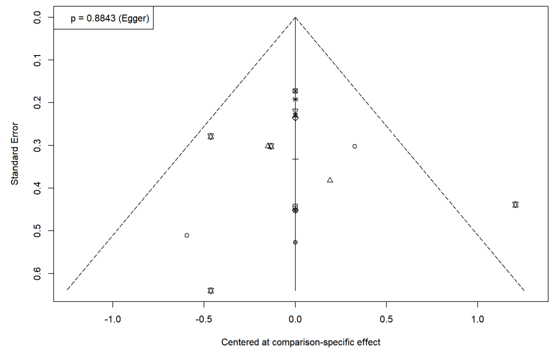

SUPPLEMENTARY MATERIAL CONTENT (SMC) 7 Funnel plots

VAS at 24 hours (after surgery)

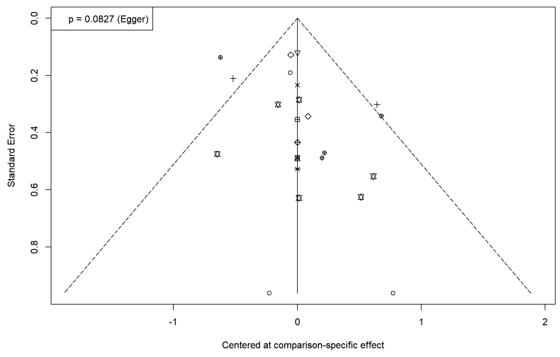

VAS at 48 hours (after surgery)

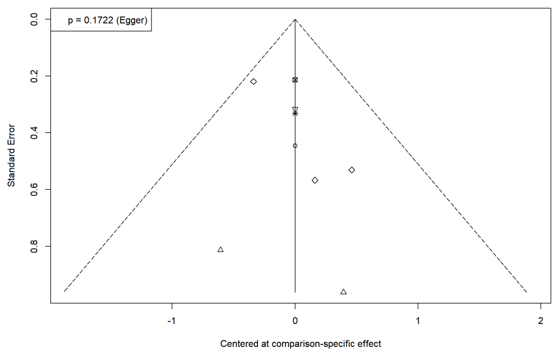

VAS at 7 days post-surgery

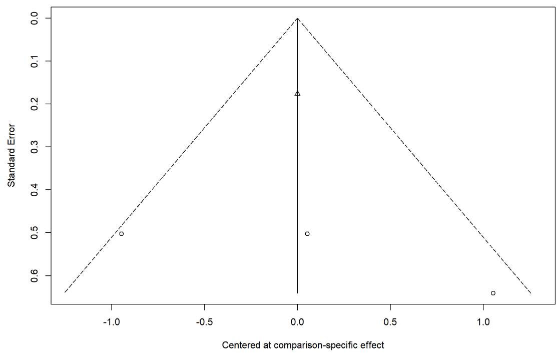

PONV

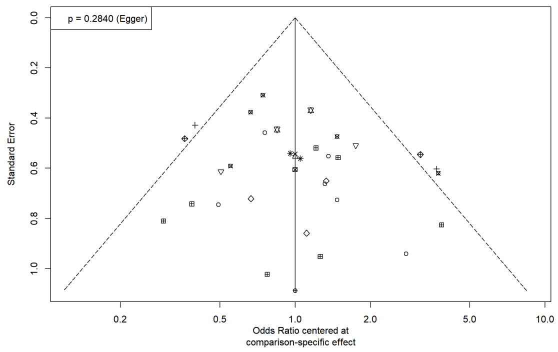

Rescue therapy during PACU stay

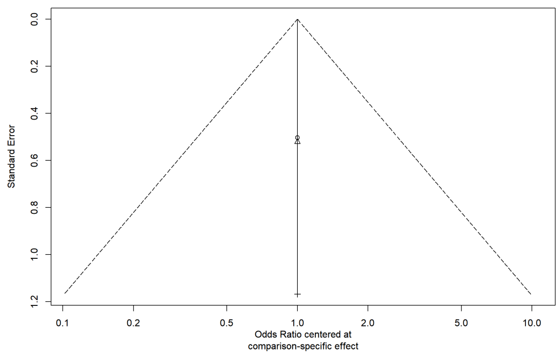

Rescue therapy within 6 hours (after surgery)

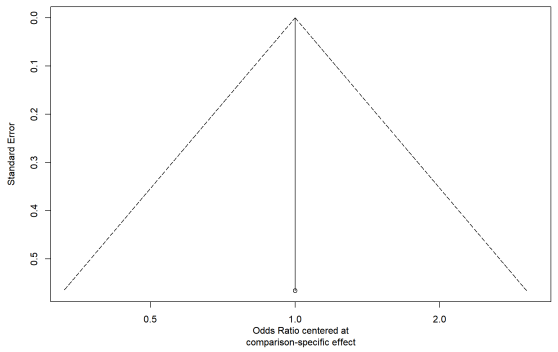

Rescue therapy within 24 hours (after surgery)

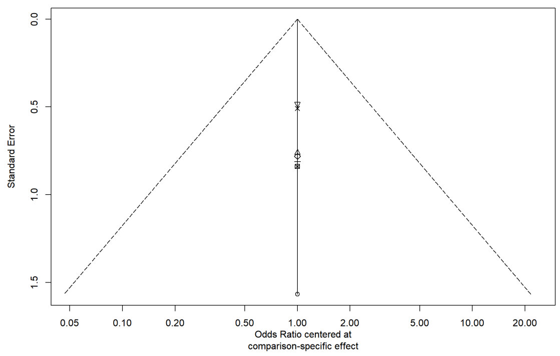

Rescue therapy within 48 hours (after surgery)

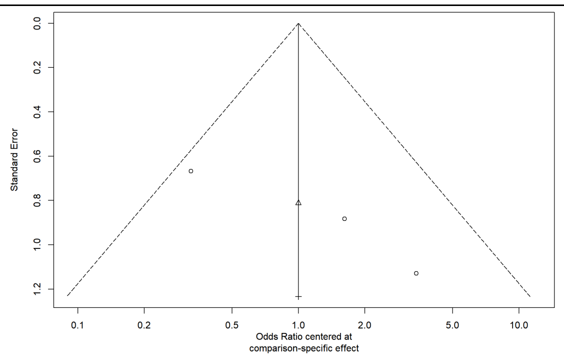

SUPPLEMENTARY MATERIAL CONTENT (SMC) 7 — Funnel plots

QoR40 on Postoperative Day 1

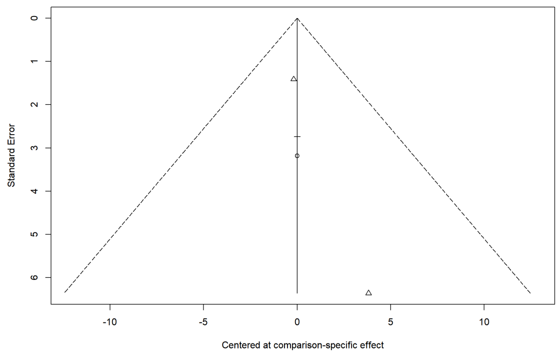

QoR40 on Postoperative Day 3

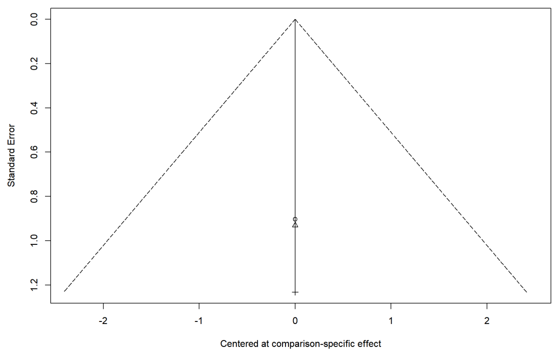

Supplement: Supplementary file 1 [file jcm-13-02100-s001.zip › SMC_JCM_R1/SMC8. Funnel plots. 04.03.24.pdf]
