# Supplementary material for: Non-Opioid Analgesics and Adjuvants after Surgery in Adults with Obesity: Systematic Review with Network Meta-Analysis of Randomized Controlled Trials
Source: J Clin Med. 2024 Apr 3;13(7):2100. doi: 10.3390/jcm13072100 (PMC11012569; doi:10.3390/jcm13072100)

Forest Plots Derived from Network Meta-Analysis for Different Time Points and Various Variables Considered

Below, a series of forest plots derived from a network meta-analysis, each corresponding to different time points and various variables considered, including Postoperative Nausea and Vomiting (PONV), Use of Rescue Analgesics, and Quality of Recovery-40 (QoR-40), are presented. Within these graphs, each study's effect size—whether it be the mean difference (MD) for continuous outcomes or the odds ratio (OR) for binary outcomes—is plotted against a central line of no effect. This line represents a value of 0 for mean differences and 1 for odds ratios, indicating no difference between treatment and control groups.

The effect sizes are accompanied by 95% confidence intervals (CIs), visually represented by horizontal lines or box-and-whisker plots that extend on either side of the point estimate. The width of these intervals provides insight into the precision of each study's estimate, with narrower intervals indicating greater precision.

The meta-analysis was conducted within a frequentist framework, employing both random and fixed effects models to account for variability within and across studies. The random effects model, which was preferred for the final analysis, assumes that the treatment effects being analyzed vary and are not exactly the same across studies, thus accommodating heterogeneity among the study outcomes.

Comparisons in this analysis were made between treatments and placebo/no intervention or other comparators, providing a comprehensive view of the relative effectiveness of interventions. The forest plots present these comparisons for each outcome of interest, offering a clear and detailed visualization of effect sizes and confidence intervals for each individual study. This facilitates an understanding of the treatment effects and their statistical significance, allowing for an informed assessment of the interventions' efficacy across different conditions and time points.

SUPPLEMENTARY MATERIAL CONTENT (SMC) 6 — Forest plots

VAS (Visual Analog Scale) at the end of surgery

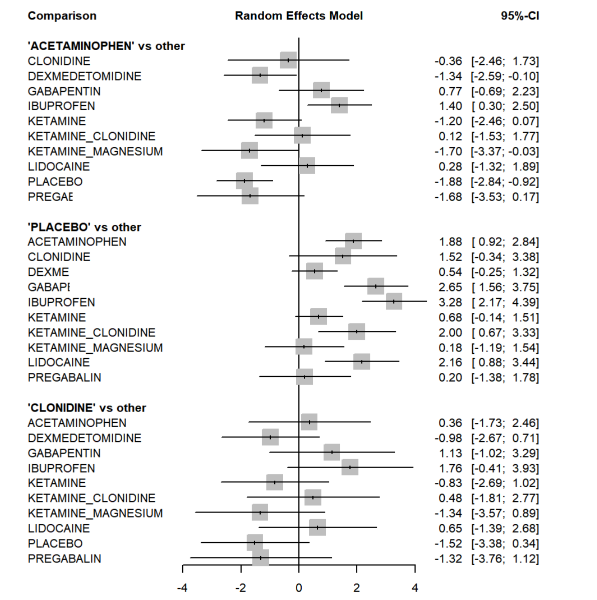

VAS at 30 minutes (after surgery)

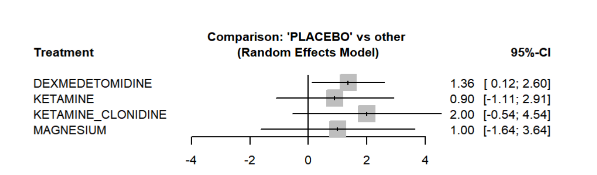

VAS at 60 minutes (after surgery)

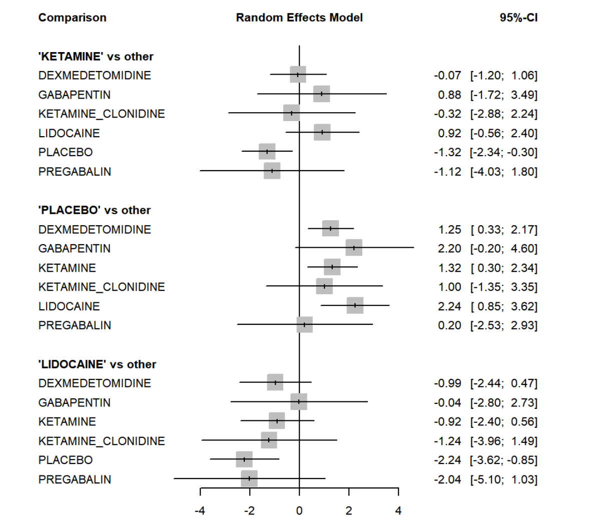

VAS at 2 hours (after surgery)

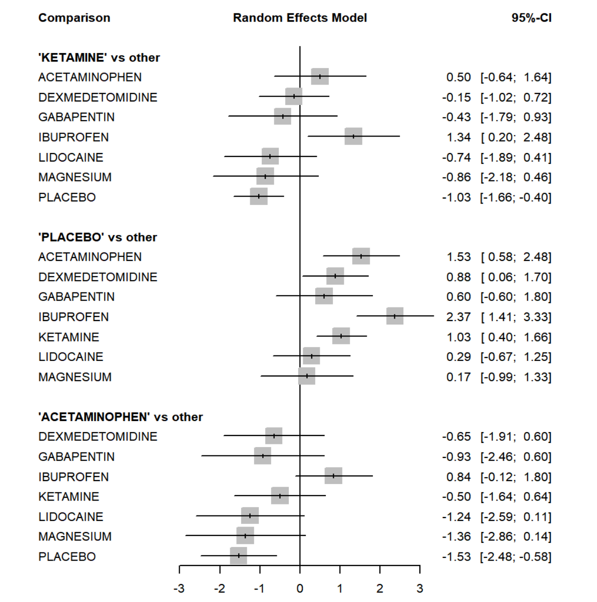

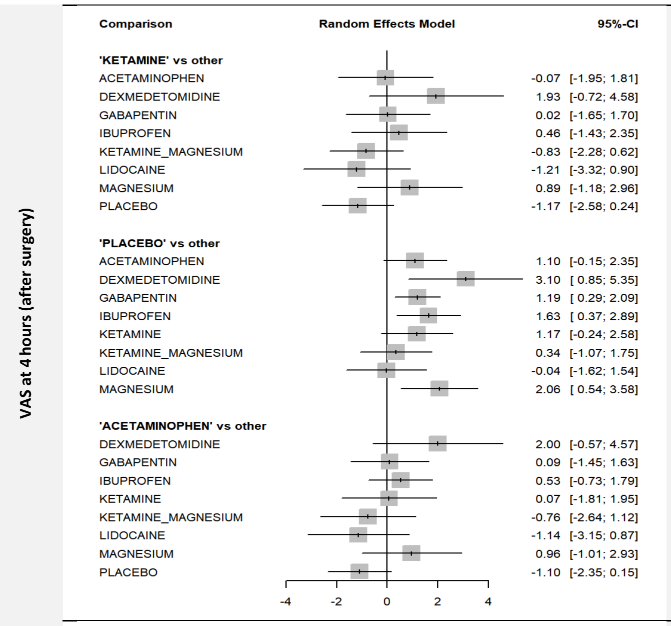
VAS at 4 hours (after surgery)
Comparison
Random Effects Model
95%-CI
'KETAMINE' vs other
ACETAMINOPHEN -0.07 [-1.95; 1.81]
DEXMEDETOMIDINE 1.93 [-0.72; 4.58]
GABAPENTIN 0.02 [-1.65; 1.70]
IBUPROFEN 0.46 [-1.43; 2.35]
KETAMINE_MAGNESIUM -0.83 [-2.28; 0.62]
LIDOCAINE -1.21 [-3.32; 0.90]
MAGNESIUM 0.89 [-1.18; 2.96]
PLACEBO -1.17 [-2.58; 0.24]
'PLACEBO' vs other
ACETAMINOPHEN 1.10 [-0.15; 2.35]
DEXMEDETOMIDINE 3.10 [0.85; 5.35]
GABAPENTIN 1.19 [0.29; 2.09]
IBUPROFEN 1.63 [0.37; 2.89]
KETAMINE 1.17 [-0.24; 2.58]
KETAMINE_MAGNESIUM 0.34 [-1.07; 1.75]
LIDOCAINE -0.04 [-1.62; 1.54]
MAGNESIUM 2.06 [0.54; 3.58]
'ACETAMINOPHEN' vs other
DEXMEDETOMIDINE 2.00 [-0.57; 4.57]
GABAPENTIN 0.09 [-1.45; 1.63]
IBUPROFEN 0.53 [-0.73; 1.79]
KETAMINE 0.07 [-1.81; 1.95]
KETAMINE_MAGNESIUM -0.76 [-2.64; 1.12]
LIDOCAINE -1.14 [-3.15; 0.87]
MAGNESIUM 0.96 [-1.01; 2.93]
PLACEBO -1.10 [-2.35; 0.15]
-4 -2 0 2 4

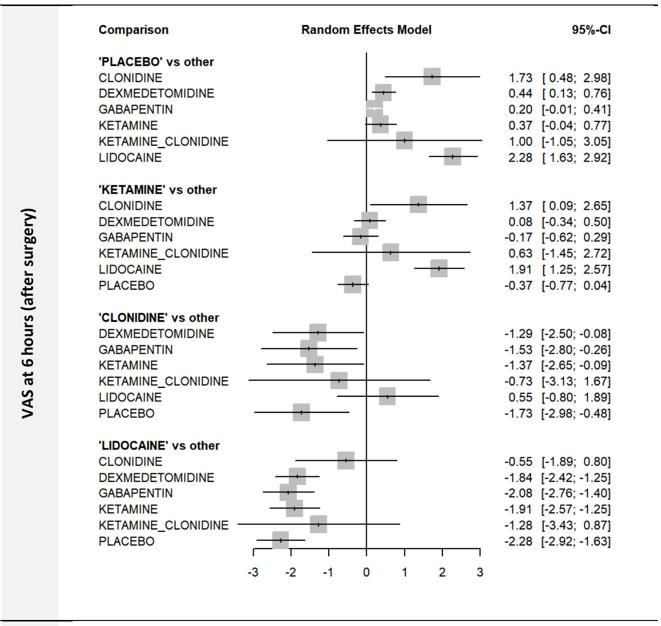
VAS at 6 hours (after surgery)
Comparison
Random Effects Model
95%-CI
'PLACEBO' vs other
CLONIDINE 1.73 [0.48; 2.98]
DEXMEDETOMIDINE 0.44 [0.13; 0.76]
GABAPENTIN 0.20 [-0.01; 0.41]
KETAMINE 0.37 [-0.04; 0.77]
KETAMINE_CLONIDINE 1.00 [-1.05; 3.05]
LIDOCAINE 2.28 [1.63; 2.92]
'KETAMINE' vs other
CLONIDINE 1.37 [0.09; 2.65]
DEXMEDETOMIDINE 0.08 [-0.34; 0.50]
GABAPENTIN -0.17 [-0.62; 0.29]
KETAMINE_CLONIDINE 0.63 [-1.45; 2.72]
LIDOCAINE 1.91 [1.25; 2.57]
PLACEBO -0.37 [-0.77; 0.04]
'CLONIDINE' vs other
DEXMEDETOMIDINE -1.29 [-2.50; -0.08]
GABAPENTIN -1.53 [-2.80; -0.26]
KETAMINE -1.37 [-2.65; -0.09]
KETAMINE_CLONIDINE -0.73 [-3.13; 1.67]
LIDOCAINE 0.55 [-0.80; 1.89]
PLACEBO -1.73 [-2.98; -0.48]
'LIDOCAINE' vs other
CLONIDINE -0.55 [-1.89; 0.80]
DEXMEDETOMIDINE -1.84 [-2.42; -1.25]
GABAPENTIN -2.08 [-2.76; -1.40]
KETAMINE -1.91 [-2.57; -1.25]
KETAMINE_CLONIDINE -1.28 [-3.43; 0.87]
PLACEBO -2.28 [-2.92; -1.63]
-3 -2 -1 0 1 2 3

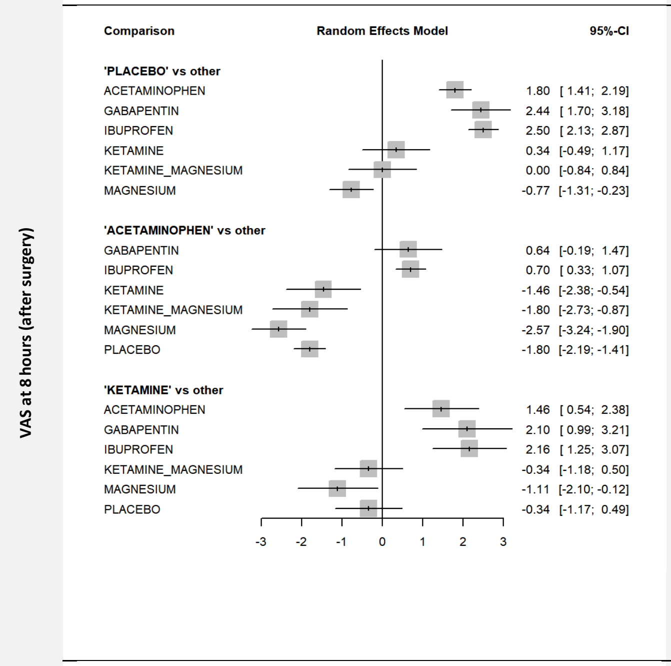
VAS at 8 hours (after surgery)
Comparison
Random Effects Model
95%-CI
'PLACEBO' vs other
ACETAMINOPHEN 1.80 [1.41; 2.19]
GABAPENTIN 2.44 [1.70; 3.18]
IBUPROFEN 2.50 [2.13; 2.87]
KETAMINE 0.34 [-0.49; 1.17]
KETAMINE_MAGNESIUM 0.00 [-0.84; 0.84]
MAGNESIUM -0.77 [-1.31; -0.23]
'ACETAMINOPHEN' vs other
GABAPENTIN 0.64 [-0.19; 1.47]
IBUPROFEN 0.70 [0.33; 1.07]
KETAMINE -1.46 [-2.38; -0.54]
KETAMINE_MAGNESIUM -1.80 [-2.73; -0.87]
MAGNESIUM -2.57 [-3.24; -1.90]
PLACEBO -1.80 [-2.19; -1.41]
'KETAMINE' vs other
ACETAMINOPHEN 1.46 [0.54; 2.38]
GABAPENTIN 2.10 [0.99; 3.21]
IBUPROFEN 2.16 [1.25; 3.07]
KETAMINE_MAGNESIUM -0.34 [-1.18; 0.50]
MAGNESIUM -1.11 [-2.10; -0.12]
PLACEBO -0.34 [-1.17; 0.49]
-3 -2 -1 0 1 2 3

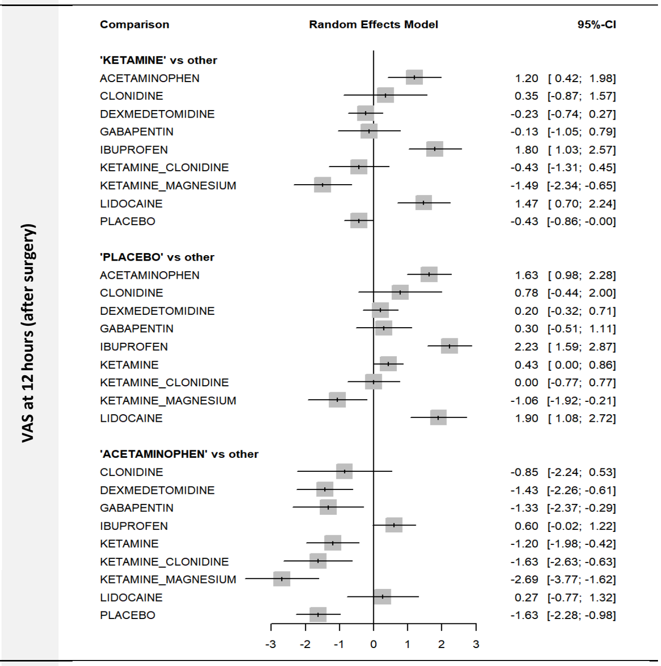
VAS at 12 hours (after surgery)
Comparison
Random Effects Model
95%-CI
'KETAMINE' vs other
ACETAMINOPHEN 1.20 [0.42; 1.98]
CLONIDINE 0.35 [-0.87; 1.57]
DEXMEDETOMIDINE -0.23 [-0.74; 0.27]
GABAPENTIN -0.13 [-1.05; 0.79]
IBUPROFEN 1.80 [1.03; 2.57]
KETAMINE_CLONIDINE -0.43 [-1.31; 0.45]
KETAMINE_MAGNESIUM -1.49 [-2.34; -0.65]
LIDOCAINE 1.47 [0.70; 2.24]
PLACEBO -0.43 [-0.86; -0.00]
'PLACEBO' vs other
ACETAMINOPHEN 1.63 [0.98; 2.28]
CLONIDINE 0.78 [-0.44; 2.00]
DEXMEDETOMIDINE 0.20 [-0.32; 0.71]
GABAPENTIN 0.30 [-0.51; 1.11]
IBUPROFEN 2.23 [1.59; 2.87]
KETAMINE 0.43 [0.00; 0.86]
KETAMINE_CLONIDINE 0.00 [-0.77; 0.77]
KETAMINE_MAGNESIUM -1.06 [-1.92; -0.21]
LIDOCAINE 1.90 [1.08; 2.72]
'ACETAMINOPHEN' vs other
CLONIDINE -0.85 [-2.24; 0.53]
DEXMEDETOMIDINE -1.43 [-2.26; -0.61]
GABAPENTIN -1.33 [-2.37; -0.29]
IBUPROFEN 0.60 [-0.02; 1.22]
KETAMINE -1.20 [-1.98; -0.42]
KETAMINE_CLONIDINE -1.63 [-2.63; -0.63]
KETAMINE_MAGNESIUM -2.69 [-3.77; -1.62]
LIDOCAINE 0.27 [-0.77; 1.32]
PLACEBO -1.63 [-2.28; -0.98]
-3 -2 -1 0 1 2 3

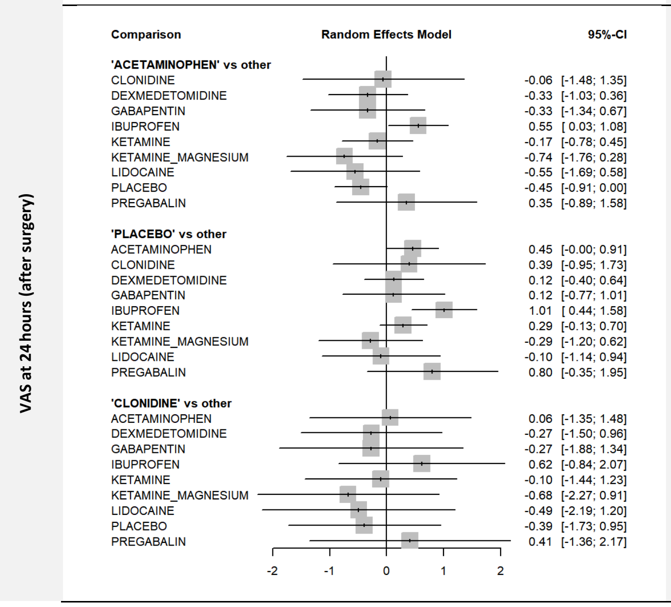
VAS at 24 hours (after surgery)
Comparison
Random Effects Model
95%-CI
'ACETAMINOPHEN' vs other
CLONIDINE -0.06 [-1.48; 1.35]
DEXMEDETOMIDINE -0.33 [-1.03; 0.36]
GABAPENTIN -0.33 [-1.34; 0.67]
IBUPROFEN 0.55 [0.03; 1.08]
KETAMINE -0.17 [-0.78; 0.45]
KETAMINE_MAGNESIUM -0.74 [-1.76; 0.28]
LIDOCAINE -0.55 [-1.69; 0.58]
PLACEBO -0.45 [-0.91; 0.00]
PREGABALIN 0.35 [-0.89; 1.58]
'PLACEBO' vs other
ACETAMINOPHEN 0.45 [-0.00; 0.91]
CLONIDINE 0.39 [-0.95; 1.73]
DEXMEDETOMIDINE 0.12 [-0.40; 0.64]
GABAPENTIN 0.12 [-0.77; 1.01]
IBUPROFEN 1.01 [0.44; 1.58]
KETAMINE 0.29 [-0.13; 0.70]
KETAMINE_MAGNESIUM -0.29 [-1.20; 0.62]
LIDOCAINE -0.10 [-1.14; 0.94]
PREGABALIN 0.80 [-0.35; 1.95]
'CLONIDINE' vs other
ACETAMINOPHEN 0.06 [-1.35; 1.48]
DEXMEDETOMIDINE -0.27 [-1.50; 0.96]
GABAPENTIN -0.27 [-1.88; 1.34]
IBUPROFEN 0.62 [-0.84; 2.07]
KETAMINE -0.10 [-1.44; 1.23]
KETAMINE_MAGNESIUM -0.68 [-2.27; 0.91]
LIDOCAINE -0.49 [-2.19; 1.20]
PLACEBO -0.39 [-1.73; 0.95]
PREGABALIN 0.41 [-1.36; 2.17]
-2 -1 0 1 2

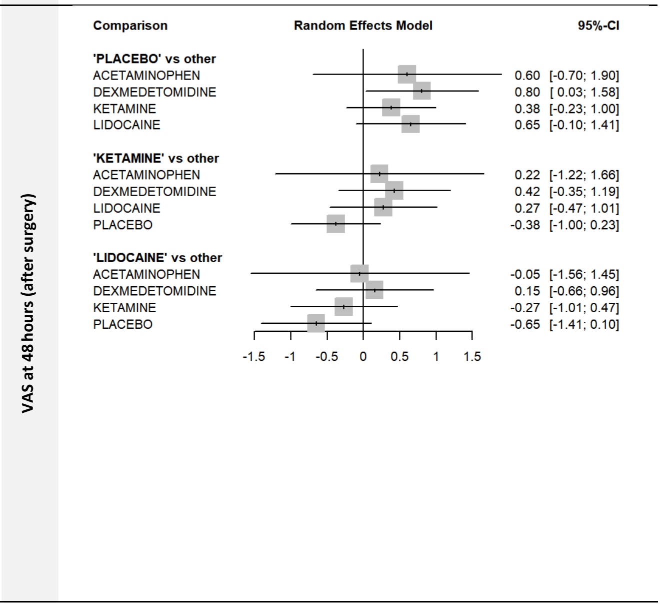
VAS at 48 hours (after surgery)
Comparison
Random Effects Model
95%-CI
'PLACEBO' vs other
ACETAMINOPHEN 0.60 [-0.70; 1.90]
DEXMEDETOMIDINE 0.80 [0.03; 1.58]
KETAMINE 0.38 [-0.23; 1.00]
LIDOCAINE 0.65 [-0.10; 1.41]
'KETAMINE' vs other
ACETAMINOPHEN 0.22 [-1.22; 1.66]
DEXMEDETOMIDINE 0.42 [-0.35; 1.19]
LIDOCAINE 0.27 [-0.47; 1.01]
PLACEBO -0.38 [-1.00; 0.23]
'LIDOCAINE' vs other
ACETAMINOPHEN -0.05 [-1.56; 1.45]
DEXMEDETOMIDINE 0.15 [-0.66; 0.96]
KETAMINE -0.27 [-1.01; 0.47]
PLACEBO -0.65 [-1.41; 0.10]
-1.5 -1 -0.5 0 0.5 1 1.5

VAS at 7 days post-surgery

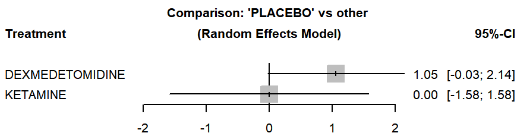

PONV

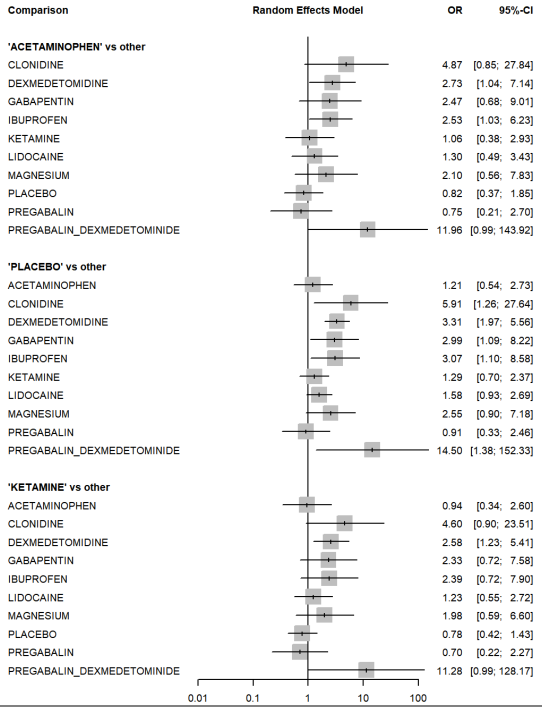

Rescue therapy during PACU stay

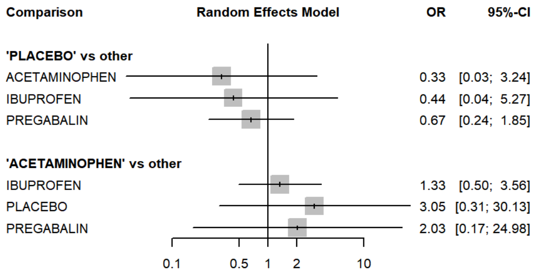

Rescue therapy within 6 hours (after surgery)

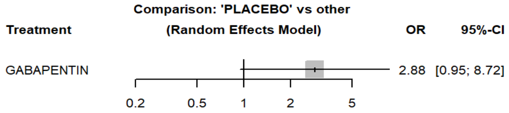

Rescue therapy within 24 hours (after surgery)

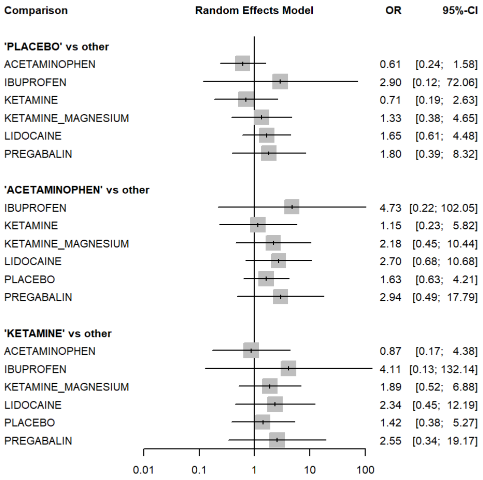

Rescue therapy within 48 hours (after surgery)

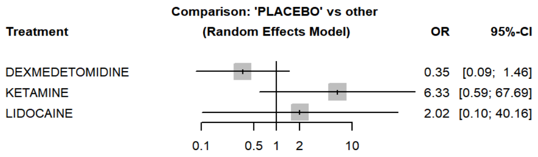

QoR40 on Postoperative Day 1

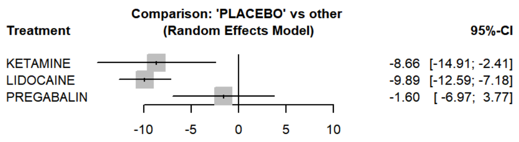

QoR40 on Postoperative Day 3

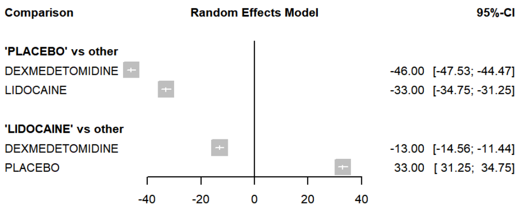

Supplement: Supplementary file 1 [file jcm-13-02100-s001.zip › SMC_JCM_R1/SMC6. Forest plots. 26.03.24.pdf]
